# Offspring Microbiomes Differ Across Breeding Sites in a Panmictic Species

**DOI:** 10.3389/fmicb.2019.00035

**Published:** 2019-02-06

**Authors:** Mark Alan Frank Gillingham, Arnaud Béchet, Frank Cézilly, Kerstin Wilhelm, Manuel Rendón-Martos, Fabrizio Borghesi, Sergio Nissardi, Nicola Baccetti, Hichem Azafzaf, Sebastian Menke, Yves Kayser, Simone Sommer

**Affiliations:** ^1^Institute of Evolutionary Ecology and Conservation Genomics, University of Ulm, Ulm, Germany; ^2^Institut de Recherche de la Tour du Valat, Arles, France; ^3^Université de Bourgogne, Equipe Ecologie Evolutive, UMR CNRS 6282 Biogéosciences, Dijon, France; ^4^Consejería de Medio Ambiente y Ordenación del Territorio, R.N. Laguna de Fuente de Piedra, Fuente de Piedra, Spain; ^5^Department of Biological Sciences, Geological and Environmental, University of Bologna, Ravenna, Italy; ^6^Anthus, s.n.c., Cagliari, Italy; ^7^Istituto Superiore per la Protezione e Ricerca Ambientale, Rome, Italy; ^8^Association “Les Amis des Oiseaux” (AAO/BirdLife Tunisie), Ariana Center, Ariana, Tunisia

**Keywords:** gut microbiome, dispersal, population differentiation, greater flamingos, *Phoenicopterus roseus*

## Abstract

High dispersal rates are known to homogenize host’s population genetic structure in panmictic species and to disrupt host local adaptation to the environment. Long-distance dispersal might also spread micro-organisms across large geographical areas. However, so far, to which extent selection mechanisms that shape host’s population genetics are mirrored in the population structure of the enteric microbiome remains unclear. High dispersal rates and horizontal parental transfer may homogenize bacterial communities between breeding sites (homogeneous hypothesis). Alternatively, strong selection from the local environment may differentiate bacterial communities between breeding sites (heterogeneous hypothesis). Furthermore, selection from age-specific environmental or physiological factors may differentiate the microbiome between juveniles and adults. Here, we analyzed the cloacal bacterial 16S rRNA gene of fledgling greater flamingos, *Phoenicopterus roseus*, across nine western Mediterranean breeding sites and four breeding seasons (*n* = 731) and adult birds (*n* = 27) from a single site. We found that fledgling cloacal microbiome, as measured by alpha diversity, beta diversity, the relative abundance of assigned sequence variants (ASVs) belonging to a phylum and genus composition within phylum, varied significantly between sampling sites and across time within site despite high adult dispersal rates. The spatio-temporal effects were stronger on individual ASV absence/presence than on ASV abundance (i.e., than on core microbiome composition). Spatial effects had a stronger effect than temporal effects, particularly on ASV abundance. Our study supports the heterogeneous hypothesis whereby local environmental conditions select and differentiate bacterial communities, thus countering the homogenizing effects of high-dispersing host species. In addition, differences in core microbiome between adult vs. fledgling samples suggests that differences in age-specific environmental and/or physiological factors result in differential selection pressure of core enteric microbiome between age classes, even within the same environment. In particular, the genus *Corynebacterium*, associated with both seasonal fat uptake and migration in previous studies, was much more abundant in high-dispersing fledglings than in more resident adults. To conclude, selection mechanisms that shape the host’s genetic structure cannot be extended to the genetic structure of the enteric microbiome, which has important implications regarding our understanding of both host local adaptation mechanisms and enteric microbiome population genetics.

## Introduction

All animals harbor a large diversity of symbiotic bacteria, particularly on mucosal surfaces such as the gut (Ley et al., 2008a,b; Kohl, 2012). An enteric microbial community in homeostasis is thought to have important fitness benefits (Shin et al., 2011; Gould et al., 2018), since symbiotic gastrointestinal bacteria are known to play an important role in facilitating nutrient intake, gut development, immune system maturation and resistance against enteric pathogens (Ley et al., 2008a,b; Stecher and Hardt, 2008; Shin et al., 2011; Phillips et al., 2012; Delsuc et al., 2014). In contrast, micro-organisms detrimental to a host, i.e., pathogens, act as a powerful selective mechanism which can shift the genetic structure of populations, cause the decline of previously thriving populations and can even be a serious threat to already vulnerable species (Altizer et al., 2003). Recent studies have focused on the role of intrinsic and extrinsic factors in shaping enteric microbiome homeostasis and how dysbiosis might affect the rise of pathogens and disease (McKenna et al., 2008; Kinross et al., 2011; Khan et al., 2012; Menke et al., 2017b). However, so far, to which extent selection mechanisms that shape host’s population genetics are mirrored in the population structure of the enteric microbiome remains unclear.

For instance, high dispersal rates between breeding sites results in high gene flow and low genetic differentiation across a species’ distribution (i.e., panmixia). However, how frequent long-distance dispersal of the host will affect the population structure of the enteric microbiome remains poorly documented. Two main hypotheses can be drawn, which we refer to as the “homogeneous hypothesis” and “heterogeneous hypothesis.” Under the homogeneous hypothesis, long-distance dispersing species may act to homogenize bacterial communities at breeding sites across large spatial scales (Finlay and Clarke, 1999; Finlay, 2002; Fenchel, 2003) and consequently environmental intake of bacterial communities between breeding sites will be similar. Alternatively, under the heterogeneous hypothesis, local environmental conditions may rapidly select for different bacterial communities countering the homogenizing effects of high-dispersing host species (Martiny et al., 2006). In this case, we predict local differentiation in enteric microbiome diversity between breeding sites. Furthermore, because sites at close proximity will often have similar environmental conditions, a positive association between breeding site distance and enteric microbiome similarity is expected.

In addition to host dispersal and selection from the environment, parental transfer of microbes from one generation to the next may also play an important role in shaping the population structure of microbiome communities. In mammals, vertical transfer via maternal effects is thought to be an important mechanism in shaping early life enteric microbiome (Palmer et al., 2007). In contrast, a recent study in arctic shorebirds demonstrated that embryos and newly hatched chicks’ guts are aseptic and that the bacterial enteric microbiome is entirely acquired post-hatching (Grond et al., 2017). In addition to similar environmental exposure, similar food provisioning and horizontal transfer may drive microbiome similarity within brood (Lucas and Heeb, 2005), since diet is known to be an important predictor of enteric microbiome diversity in many organisms (Muegge et al., 2011). However, juveniles may also have very different adaptive needs during growth and development from adults and age-specific selection pressure may be differentiating core microbiome composition between adults and juveniles (Yatsunenko et al., 2012; Markle et al., 2013; Saraswati and Sitaraman, 2015; Odamaki et al., 2016). Therefore, under the homogeneous hypothesis, horizontal transfer from parents will result in a similar microbiome community between juveniles and adults. The latter mechanism will further homogenize microbiome communities of juveniles between breeding sites. Thus, under the homogeneous hypothesis we expect little differentiation in fledgling enteric microbiome communities between breeding sites and between adults and fledglings. Alternatively, under the heterogeneous hypothesis, due to strong differences in life-history traits between fledglings and adults, strong differential selection pressure of adaptive enteric microbiome and thus strong differentiation in microbiome between adults and fledglings, even within the same environment, is expected.

Colonial waterbirds are an ideal model to investigate the effect of long-distance dispersal on enteric microbiome. Highly mobile and often performing long-distance movements, they can spread micro-organisms across large geographical areas, especially through their feces (Altizer et al., 2011). Furthermore, the large congregation of individuals during the breeding season means that colonial waterbirds use extensively fecal contaminated waterbodies (Klimaszyk and Rzymski, 2013; Telesford-Checkley et al., 2017), and are thus prone to involuntary coprography. Therefore, the transfer of both non-pathogenic and pathogenic microorganisms between conspecific hosts is likely to be highly facilitated in colonial birds (Rifkin et al., 2012). Investigation of 16S rRNA gene sequences in waterbird feces has revealed a high prevalence of waterborne gastrointestinal pathogenic strains of *E. coli, Campylobacter* and *Salmonella* (Lu et al., 2011; e.g., Waldenström et al., 2002; Wetzel and LeJeune, 2007; Vogel et al., 2013; Telesford-Checkley et al., 2017).

In this study, we used the Greater Flamingo, *Phoenicopterus roseus*, as a biological model to examine spatial variation in enteric microbiome in a long-lived colonial waterbird species. Previous work has provided evidence for panmixia across all breeding colonies at the scale of the Mediterranean (Geraci et al., 2012; Gillingham et al., 2017), as a result of high dispersal rates (Barbraud et al., 2003; Balkız et al., 2010; Sanz-Aguilar et al., 2012; Gillingham et al., 2013). We investigated the cloacal bacterial microbiome of fledglings (i.e., juvenile individuals about to take flight but still fed by parents) using 16S rRNA gene sequencing as a non-invasive proxy of enteric microbiome at nine Mediterranean breeding colonies across four breeding seasons. Furthermore, in order to compare the cloacal bacterial microbiome of fledgling individuals to that of mature adults, we also analyzed adult samples within the same site and year as one single fledglings’ sampling site.

In this study, we therefore tested whether:

(1) Fledgling cloacal bacterial communities, including potential pathogens, were differentiated between breeding colonies across the Mediterranean, despite high host dispersal rates and gene flow;

(2) Fledgling cloacal bacterial communities, including potential pathogens, were differentiated between years within the same site;

(3) Fledgling and adult bacterial communities were differentiated within the same site and year.

## Materials and Methods

### Study Species

Greater flamingos congregate in large numbers and are the most abundant species in terms of biomass in Mediterranean wetlands (Johnson and Cézilly, 2007). They specialize in filter feeding of invertebrates and seeds in brackish wetlands and salt pans (Johnson and Cézilly, 2007). During filter feeding they can ingest a considerable quantity of sediments (Jenkin, 1957), making them vulnerable to environmental contaminants (Borghesi et al., 2016), to involuntary coprography and to bacterial exposure of extensively fecal contaminated waterbodies (Benskin et al., 2009). During chick rearing, adults feed their young with a liquid secreted from the upper digestive tract, rich in proteins, fat, carotenoids and blood cells (Lang, 1963; Fisher, 1972), and presumably transfer parental bacteria. Furthermore, greater flamingos have high dispersal rates across major Mediterranean wetland habitats (Barbraud et al., 2003; Balkız et al., 2010; Sanz-Aguilar et al., 2012; Gillingham et al., 2013). They are therefore likely to be an important vector of bacterial dispersal and are likely to play a major role in shaping bacterial communities between Mediterranean wetlands. Previous studies, have found no genetic differentiation at either neutral (microsatellites and mitochondria) (Geraci et al., 2012) or functional markers (major histocompatibility genes involved in the recognition of antigens within the innate immune system) (Gillingham et al., 2017) between breeding colonies across Mediterranean colonies.

**FIGURE 1:**
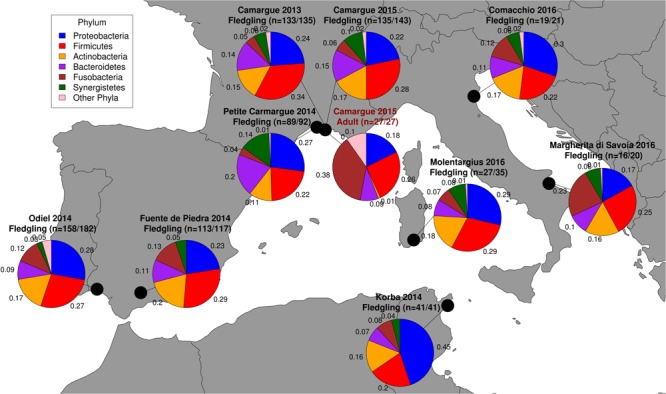
Sampling locations of the nine greater flamingo breeding sites. Sample sizes are in the header of sampling unit name and the first number indicates the number of samples successfully sequenced and the second, the number of samples collected. Pie charts represent population level enteric microbiome composition of the six most dominant phyla, and numbers next to pie charts represent the relative abundance of ASV belonging to a phylum.

Breeding sites represent a diverse set of wetland habitats, ranging from man-managed salt pans, to natural lagoons and shallow tidal mudflats. Breeding in most sites is irregular and dependent on favorable climatic conditions and local water levels (Cézilly et al., 1995; Johnson and Cézilly, 2007; Béchet and Johnson, 2008; Béchet et al., 2009, 2012). The most stable and productive breeding colony in the western Mediterranean is in the Camargue (Figure 1), southern France, which is a saturated site. Since 1974 greater flamingos have bred on a small artificial island (≈4,000 m^2^) with an average of 10,000 breeding pairs. In Spain, the Fuente de Piedra lagoon (Figure 1) hosts the second most important breeding site in the western Mediterranean, although flamingos may fail to breed there in years of low rainfall (occurring about 50% of the time in the last 50 years) (Rendón et al., 2001; Balkız et al., 2010). However, in favorable years, the size of the breeding colony frequently exceeds that of the one in southern France (with up to 20,000 breeding pairs). The remaining sampling sites in this study are smaller colonies that have been established recently (from 1993 onward; Johnson and Cézilly, 2007). Of these new sites, the semi-natural lagoon bordered by the urban areas of Cagliari on the west and Quartu on the east in Sardinia, Italy (Molentargius, Figure 1) has been the most stable and largest since 2006.

### Sample Collection

We collected 814 cloacal swabs of greater flamingo fledglings during ringing operations (Johnson and Cézilly, 2007) at nine breeding sites across the western Mediterranean basin between 2013 and 2016 (Figure 1): the Fangassier’s lagoon (Figure 1; 43°25′N, 4°37′E; Salin-de-Giraud, Camargue, France) in 2013 (Camargue 2013; *n* = 135) and 2015 (Camargue 2015 *n* = 143); Étang du Roi (Figure 1; 43°31′N, 4°12′E; Aigues-Mortes, Petite Camargue, France) in 2014 (Petite Camargue 2014; *n* = 92); Fuente de Piedra lagoon (Figure 1; 37°06′N, 4°45′W; Fuente de Piedra, Málaga, Spain) in 2014 (Fuente de Piedra 2014; *n* = 117); Odiel marshes (Figure 1; 37°12′N, 6°58′W; Huelva, Andalucía, Spain) in 2014 (Odiel 2014; *n* = 182); Korba lagoon (Figure 1; 36°35′N, 10°52′E; Korba, Nabeul Governorate, Tunisia) in 2014 (Korba 2014; *n* = 41); Comacchio (Figure 1; 44°39′N, 12°10′E; Comacchio, Province of Ferrara, Italy) in 2016 (Comacchio 2016; *n* = 21); Molentargius saline (Figure 1; 39°13′N, 9°09′E; Cagliari, Sardinia, Italy) in 2016 (Molentargius 2016; *n* = 35); and, Margherita di Savoia salines (Figure 1; 41°22′N, 16°09′E, Barletta-Andria-Trani Puglia, Italy) in 2016 (Margherita di Savoia 2016; *n* = 20). Since the Fangassier’s lagoon was sampled twice, we refer to year and site specific sampling events as sampling units. Cloacal swabs were collected using forensic swabs (Sarstedt, Nuembrecht, Germany) that were placed in 2 ml Eppendorf tubes with 600 μl of RNA*later^®^*. All samples were stored at -20°C within 48 h of sampling until DNA extraction was performed. Fledglings were 2–4 months at the time of sampling.

In 2015, we additionally sampled 25 adult fecal samples and two cloacal swabs from dead adult birds at the Pont-de-Gau ornithological park (Figure 1; 43°29′N, 4°24′E; Saintes-Maries-de-la-Mer, Camargue, France). There, wild flamingos are attracted by regular food provisioning, such that the birds are habituated to close human contact. Flamingos observed at Pont de Gau are likely to be the parents of chicks from the nearby Fangassier breeding colony in the Camargue. It was therefore possible to directly compare the enteric microbiome of adults with that of fledglings within the same environmental setting by using the fledgling and adult samples collected in 2015 in the Camargue. Fecal samples of adults were collected by observing at a distance whether a bird crossed and defecated on a dike between two ponds, at the time of the day Pont-de-Gau ornithological park staff feed wild birds with rice. Fresh samples were collected immediately using swabs and environmental soil contamination was avoided by sampling at the core of the feces. Additional cloacal swabs were collected from freshly (<24 h) dead birds (whose corpses had been immediately frozen after delivery to staff at the Pont-de-Gau ornithological park), and stored as previously described.

### DNA Extraction of Swabs

Following the protocol detailed in Menke et al. (2017a), 200 μl of our samples were mixed with 1 ml of InhibitEx buffer and homogenized with ceramic beads for 2 × 3 min on a SpeedMill (Analytik Jena, Germany). Thereafter, we followed the manufacturer’s protocol for DNA extraction of stool samples using the Qiagen Cador Pathogen extraction kit (Qiagen, Hilden, Germany). During extraction we included 41 blanks whereby only the reagents used during extraction were extracted.

### PCR Amplification, Library Preparation, and High-Throughput Sequencing

We amplified a 291-bp fragment of the hypervariable V4 region of the 16S rRNA gene using the universal bacterial primers 515F (5′-GTGCCAGCMGCCGCGGTAA-3′) and 806R (5′-GGACTACHVGGGTWTCTAAT-3′) (Caporaso et al., 2010, 2011). Individual PCR reactions were tagged with a 10-base pair identifier, using a standardized Fluidigm protocol (Access Array^TM^ System for Illumina Sequencing Systems, ©Fluidigm Corporation). For this protocol, we first did a target specific PCR using the primer 515F appended with the CS1 adapter (CS1-TS-515F) and the 806R primer appended with the CS2 adapter (CS2-TS-806R). To enrich base pair diversity of our libraries during cluster identification, we added four random bases to our forward primer. The CS1 and CS2 adapters were then used in a second PCR to add the 10 bp barcode sequence and the adapter sequences used by Illumina during sequencing.

The first PCR consisted of 3–5 ng of extracted DNA, 0.5 units FastStart Taq DNA Polymerase (Roche Applied Science, Mannheim, Germany), 1x PCR buffer, 4.5 mM MgCl_2_, 250 μM each dNTP, 0.5 μM primers, and 5% dimethylsulfoxide (DMSO). The PCR was carried out with an initial denaturation step at 95°C for 4 min followed by 30 cycles at 95°C for 30 s, 60°C for 30 s, 72°C for 45 s, and a final elongation step at 72°C for 10 min. The second barcoding PCR consisted of 1.5 μl of the first PCR product, 1 unit FastStart Taq DNA Polymerase, 1x PCR buffer, 4.5 mM MgCl2, 200 μM each dNTP, and 80 nM per barcode primer. PCR conditions were identical to the first PCR, except that amplification was reduced to 10 cycles. Amplifications were quantified by UV/VIS spectroscopy on the Xpose (Trinean, Gentbrugge, Belgium) and samples were pooled to equimolar amounts of DNA. The library was prepared as recommended by Illumina (Miseq System Denature and Dilute Libraries Guide 15039740 v05) and was loaded at 7.5 pM on a MiSeq flow cell with a 10% PhiX spike. Paired-end sequencing was performed over 2 × 251 cycles. We ran our 814 samples, 41 extraction blanks and 44 PCR blanks (a total of 85 extraction and PCR controls) across five sequencing runs.

### Bioinformatics

We used DADA 2 (Callahan et al., 2016) within QIIME 2^1^ (Caporaso et al., 2010) to denoise our data from artifacts (including chimeras), merge paired-end sequences and remove primer sequences. Unlike traditional methods of clustering sequences into operational taxonomic units (OTUs) according to a fixed threshold, DADA 2 denoises datasets to resolve sequence variants that differ by as little as one nucleotide and are referred to as assigned sequence variants (ASVs) (Callahan et al., 2016, 2017). Such methods are now recommended since, among other benefits, they provide reliable datasets with higher resolution (see Callahan et al., 2017). We did further data filtering by removing any ASV with fewer than 20 sequences in the entire dataset and excluded samples with fewer than 10,000 sequences. We assigned ASVs to taxonomy within QIIME 2 using the Greengenes database (DeSantis et al., 2006) and excluded any sequences that did not assign to any known bacterial lineage at the phylum taxonomic level. A tree was built using FastTree 2.1.8 (Price et al., 2010). A archaea sequence (accession number: KU656649) was used to root the tree. The branch and taxonomic name of the root archaea sequence was removed prior to analysis. We imported our data (only samples with at least 10,000 sequences) and the rooted FastTree into R version 3.4.4 (R Core Team, 2018) using the R package “phyloseq” (McMurdie and Holmes, 2013). All sequences that were found with a relative abundance of least 1% and found in at least three samples across extraction and PCR blanks were considered to be contaminant sequences and were excluded from the dataset.

### Statistics

We calculated Faith’s phylogenetic diversity (PD) for each sample as a measure of alpha diversity (Faith, 1992) using the btools R package (Battaglia, 2018). We first investigated whether alpha diversity (PD) of the microbiome differed between sampling units and age class (fledgling vs. adult samples) controlling for sample sequencing depth using a General Additive Model (GAM), with a Gamma error distribution and a log link function (since PD distribution behaved similarly to count data but was continuous) using the mgcv package (Wood, 2006). Sampling depth was fitted as a smoother. Model selection was achieved through information-theoretic (I–T) model selection (Burnham and Anderson, 2002). All possible candidate models were constructed and Akaike’s Information Criterion adjusted for small sample sizes (AICc) and AICc weights (ω) were used to assess the relative strength of support for models (Burnham and Anderson, 2002). We also report the adjusted *R*^2^ of each GLM model as defined by the MuMIn R package (Bartón, 2016). We also report the odds ratio effect size and 95% confidence intervals (Nakagawa and Cuthill, 2007).

We then investigated whether beta diversity of the microbiome differed between sampling units and age class. Because beta diversity analyses are sensitive to variation of rare ASVs, we excluded ASVs which occurred in only 10 individuals or less. For this analysis, we separated our dataset into two smaller datasets in order to partition the variance explained by each explanatory variable. The samples from fledglings (excluding adult samples) were treated as one dataset and samples from Camargue in 2015 (fledgling and adult samples) were treated as a second set. For each dataset, we calculated the weighted and unweighted UniFrac metric (Lozupone and Knight, 2005), using the R package “phyloseq.” We used weighted as well as unweighted UniFrac metrics to discriminate the effect our explanatory variables had on ASV relative abundances (weighted UniFrac), i.e., core microbiome composition, from ASV absence/presence (unweighted UniFrac), i.e., microbiome composition irrespective of ASV abundance. Log likelihoods, AICc and *R*^2^ values of Permutational Multivariate Analysis of Variance (PERMANOVA) models (9,999 permutations) were calculated [adonis function in R package “vegan” (Oksanen et al., 2018)]. Model selection was achieved through information-theoretic (I–T) model selection as described above. In order to visualize results from the PERMANOVA analyzes, we plotted the centroids and associated standard errors of the first three axis of principle coordinates analyzes (PCoA) (in the Supplementary Material, we also present PCoA figures of the raw data with ellipses representing 95% confidence intervals; Supplementary Figures S3, S4). To estimate effect size of explanatory variables on beta diversity, Cohen’s *d* (Cohen, 1988) of the difference between each pairs of centroids of sampling units for each of the first three PCoA axes were also calculated and presented in the Supplementary Table S3.

To estimate the variance explained by temporal variation independently of any spatial effects, we repeated the above weighted and unweighted UniFrac analyses with a dataset containing only fledgling samples collected in the Camargue (2013 and 2015). Similarly, to estimate the variance explained by spatial variation independently of any temporal effects, we repeated the weighted and unweighted UniFrac analyses with a dataset containing only fledgling samples collected in 2014 at four different locations (Petite Camargue, Fuente de Piedra, Odiel and Korba). Model selection for the latter two analyses are presented in the Supplementary Table S4.

Variation in microbiome between breeding sites may be explained by variation in fledgling age between breeding sites. Consequently, we repeated analyses on alpha and beta diversity controlling for tarsus length, a very good proxy of fledgling age in tall, lengthy greater flamingos (Johnson and Cézilly, 2007). Model selection for the latter analyses are presented in the Supplementary Tables S1, S2.

The relative abundance of ASVs belonging to a phylum was plotted at the sampling unit level. The relative abundance of ASVs belonging to a phylum according to sampling unit for the six most common phyla (Proteobacteria, Firmicutes, Actinobacteria, Bacteroidetes, Fusobacteria, and Synergistetes) was modeled using a GLM with a binomial error distribution and a logit link function. Model selection was achieved as described above for previous analyses but using quasi-AICc (QAICc) as an information criterion to control for overdispersion. We also report the odds ratio effect size and 95% confidence intervals (Nakagawa and Cuthill, 2007). In addition, we investigated genus composition of each phyla at the sampling unit level.

Plots were created by either using the core R software or by using the R package “ggplot 2” (Wickham, 2016).

## Results

### Raw Data of Sequencing Run

Following data quality filtering, 20,775,119 sequences from 758 samples out of 814 (93% of extracted samples) remained in our dataset (BioProject PRJNA485732; Accession number: SAMN10576470–SAMN10577345). From these 758 samples, we found 6,957 ASVs. All 85 extraction and PCR blanks had fewer than 10,000 sequences in total and were not retained after data quality filtering. Removing sequences that were found with a relative abundance of least 1% and found in at least three samples across extraction and PCR blanks resulted in the removal of 15 ASVs (therefore 6,942 ASVs remained in the datasets after the removal of contaminant ASVs, Supplementary Figure S1).

### Alpha Diversity

**FIGURE 2:**
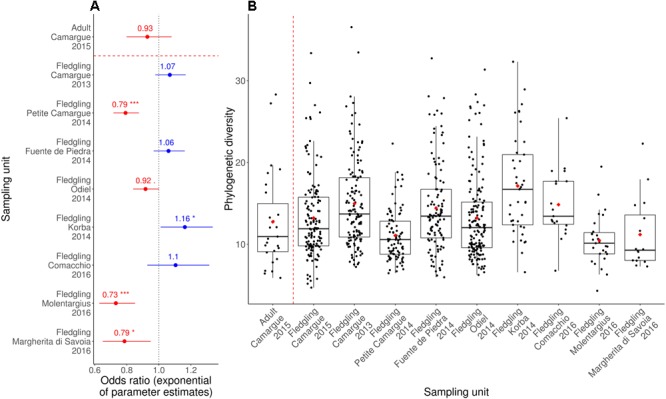
**(A)** Odds ratio (exponential of parameter estimates) of the GLM (with a Gamma distribution and log link function) of phylogenetic diversity according to sampling unit. The intercept (dotted black line) is fledgling samples from Camargue in 2015. Odds ratio values are displayed as well as significance relative to the intercept (^∗^*p* = 0.05, ^∗∗^*p* = 0.01, ^∗∗∗^*p* < 0.001). **(B)** Boxplot and mean (red diamonds) of phylogenetic diversity according sampling unit. Data points are jittered to indicate sample size. The dashed red line separates adult samples from fledgling samples.

Model selection strongly supported an effect of sampling unit on PD (Table 1; ΔAIC = 70.03). Furthermore, odds ratios and 95% confidence intervals revealed significant differences in PD between sampling units (Figure 2). Within the same sampling site of Camargue, PD was not significantly different between 2013 and 2015 (Figure 2). There was low support for a difference in PD between fledgling samples and adult samples (Table 1; ΔAIC = 0.52), with equivalent odd ratios found for samples collected from fledglings and adults in Camargue in 2015 (Figure 2). Controlling for differences in fledgling age did not quantitatively change the results, except for the Korba 2014 sampling unit which was no longer significantly higher than Camargue 2015 (see Supplementary Tables S1, S2 and Supplementary Figure S2).

**Table 1:** Model selection of GAMs (with a Gamma distribution and log link function) of phylogenetic diversity (PD) according to sampling unit and age class (adults vs. fledgling).

Model rank	Sequencing depth	Fledgling sampling unit	Age class	d.f.	logLik	AICc	Δ AICc	AICc ω	*R*^2^
1	+	+	+	13	–2188.538	4404.5	0.00	0.564	0.148
2	+	+		12	–2189.870	4405.0	0.52	0.436	0.145
3		+		9	–2213.114	4444.5	39.96	<0.001	0.090
4		+	+	9	–2212.128	4444.6	40.04	<0.001	0.093
5	+			4	–2232.546	4474.5	70.03	<0.001	0.042
6	+		+	5	–2232.257	4476.1	71.56	<0.001	0.043
7				1	–2248.932	4501.9	97.37	<0.001	0.000
8			+	3	–2248.548	4503.1	98.61	<0.001	0.001

*The parameters included in the model are indicated by “+” and degrees of freedom (d.f.), log likelihood (logLik), Akaike Information Criterion for small sample sizes (AICc), delta AICc (Δ AICc), AIC weight (AICc ω), and adjusted-*R*^2^ are shown. Models are ranked according to model support (from the smallest AICc to the largest). The smoother was applied to the parameter sample sequencing depth.*

### Beta Diversity

**FIGURE 3:**
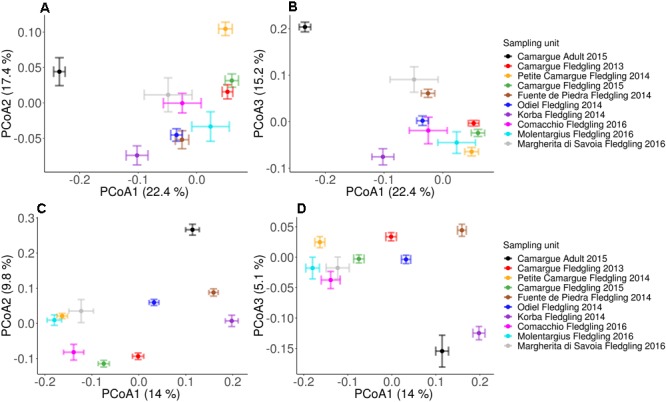
Principle coordinates analysis (PCoA) plots according to: sampling unit for PCoA axis 1 and 2 **(A)** and PCoA axis 1 and 3 **(B)** based on the weighted Unifrac distance; and sampling site for PCoA axis 1 and 2 **(C)** and PCoA axis 1 and 3 **(D)** based on the unweighted Unifrac distance. Squares represent centroids, and bars are standard errors.

PERMANOVA model selection strongly supported an effect of fledgling sampling unit on beta diversity when using both the weighted (Table 2a.; ΔAIC = 77.11; Figure 3 and Supplementary Figure S3) and unweighted UniFrac (Table 2b.; ΔAIC = 138.89; Figure 3 and Supplementary Figure S4) distance matrices. On average the effect size (using fledgling sampling units only) was significantly higher when using the unweighted UniFrac distance matrix compared to the weighted UniFrac distance matrix (mean [±95% CI]; unweighted UniFrac distance matrix: PCoA axis 1 = 1.475 [1.091; 1.860]; PCoA axis 2 = 0.814 [0.639; 0.989]; PCoA axis 3 = 0.667 [0.482; 0.852]; weighted UniFrac distance matrix: PCoA axis 1 = 0.523 [0.402; 0.644]; PCoA axis 2 = 0.613 [0.461; 0.766]; PCoA axis 3 = 0.653 [0.504; 0.802], but see Cohen’s D of difference in centroids and associated 95% CI between each pair of fledgling sampling unit for each PCoA axis presented in Supplementary Table S3). Taken together, the latter results suggest that spatio-temporal variation had a stronger effect on ASV absence/presence than on ASV abundance. Controlling for differences in fledgling age did not quantitatively change the results (see Supplementary Table S2).

**Table 2:** Model selection of PERMANOVA according to fledgling sampling unit using a weighted UniFrac distance matrix (a) and an unweighted Unifrac distance matrix (b) and according to age class (adults vs. fledgling) for a weighted UniFrac distance matrix (c) and an unweighted Unifrac distance matrix (d).

Model rank	Fledgling sampling unit	Age class	d.f.	logLik	AICc	Δ AICc	AICc ω	*R*^2^
**a. Dataset with fledgling samples only; weighted UniFrac**					
1	+		9	–69.994	180.2	0.00	1.000	0.14
2			1	–124.672	257.3	77.11	<0.001	0.00
**b. Dataset with fledgling samples only; unweighted UniFrac**					
1	+		9	–349.406	739.1	0.00	1.000	0.19
2			1	–426.972	861.9	138.89	<0.001	0.00
**c. Dataset with fledgling and adult samples from 2015 only; weighted UniFrac**					
1		+	2	117.497	–222.9	0.00	1.000	0.26
2			1	93.184	–178.3	46.58	<0.001	0.00
**d. Dataset with fledgling and adult samples from 2015 only; unweighted UniFrac**					
1		+	2	58.705	–105.3	0.00	1.000	0.24
2			1	35.970	–67.9	43.42	<0.001	0.00

*The parameters included in the model are indicated by “+” and degrees of freedom (d.f.), log likelihood (logLik), Akaike Information Criterion for small sample sizes (AICc), delta AICc (Δ AICc), AIC weight (AICc ω), and *R*^2^ are shown. Models are ranked according to model support (from the smallest AICc to the largest).*

Within sites that were sampled within the same year (hence controlling for any temporal effects), the two breeding sites at close proximity in 2014, Fuente de Piedra and Odiel, clustered closely together on the first and second PCoA axes when using the weighted UniFrac distance matrix (Figure 3, Supplementary Figure S3, and Supplementary Table S3). However, when using unweighted UniFrac, we found stronger differences in beta diversity between Fuente de Piedra 2014 and Odiel 2014 across all three PCoA axes (Figure 3, Supplementary Figure S4, and Supplementary Table S3). Regardless of the UniFrac distance matrix, Petite Camargue 2014 and Korba 2014 clustered distantly from each other and from Fuente de Piedra 2014 and Odiel 2014 (Figure 3 and Supplementary Figure S3). Reducing the dataset to samples collected in 2014, we found that spatial variation explained 14% and 18% of the variation when using weighted and unweighted UniFrac distance matrices, respectively (Supplementary Table S4).

When using the weighted UniFrac distance matrix, the paired sampling units that were the most similar across all three PCoA axes were Camargue Fledgling 2013 and Camargue Fledgling 2015 (Figure 3, Supplementary Figure S3, and Supplementary Table S3). However, when using the unweighted UniFrac distance matrix, we found stronger differences in beta diversity between Camargue Fledgling 2013 and Camargue Fledgling 2015 (Figure 3, Supplementary Figure S3, and Supplementary Table S3). The latter suggests a stronger temporal effect on beta diversity for ASV absence/presence than for ASV abundance in the Camargue. When repeating the analysis using a subsetted dataset containing samples collected in Camargue only (thus controlling for spatial effects), we found that temporal effects explained 4% and 8% of the variation when using weighted and unweighted UniFrac distance matrices, respectively (Supplementary Table S4). Taken together our results therefore suggests a much a weaker effect of temporal variation than spatial variation on beta diversity.

PERMANOVA model selection strongly supported an effect of age class on beta diversity when using both the weighted (Table 2c.; ΔAIC = 46.58; Figure 3 and Supplementary Figure S3) and unweighted UniFrac (Table 2d; ΔAIC = 43.42; Figure 3 and Supplementary Figure S4) distance matrices. The effect of age class on beta diversity had a very strong effect as revealed by the first PCoA axis when using the weighted UniFrac (Camargue Adult 2015–Camargue Fledgling 2015; Cohen’s D [95% CI]; PCoA axis 1 = 2.400 [1.994; 2.807]) and the second axis when using the unweighted UniFrac (Camargue Adult 2015–Camargue Fledgling 2015; Cohen’s *d* [95% CI]; PCoA axis 1 = 3.601 [3.100; 4.103]) distance matrices. Most of the adult samples (25 out of 27) were fecal swabs rather than cloacal swabs and two samples were cloacal swabs from freshly deceased individuals. When visualizing a PCoA according to age class and sample type (fecal swabs vs. cloacal swabs), samples clustered according to age class rather than sample type when using the weighted Unifrac distance matrix (Supplementary Figure S3). A similar amount of variation was explained by age class using the weighted and the unweighted UniFrac distance matrices (26% and 24%, respectively). However, the clustering according to age class rather than sample type was less clear when using the unweighted Unifrac distance matrix (Supplementary Figure S4).

### Relative Abundance of Bacterial Phyla

**FIGURE 4:**
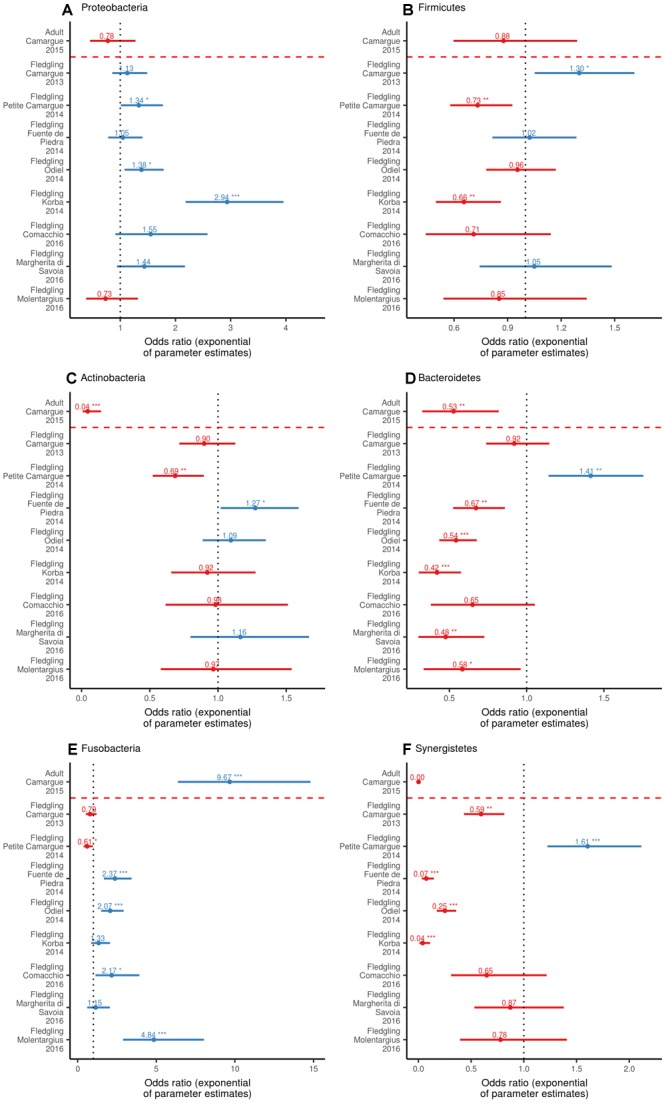
Odds ratio (exponential of parameter estimates) of the GLM (with a Binomial distribution and logit link function) of the relative abundance of ASV belonging to phylum within individual according to sampling unit for Proteobacteria **(A)**, Firmicutes **(B)**, Actinobacteria **(C)**, Bacteroidetes **(D)**, Fusobacteria **(E)**, and Synergistetes **(F)**. The intercept (dotted black line) is fledgling samples from Camargue in 2015. Odds ratio values are displayed as well as significance relative to the intercept (^∗^*p* = 0.05, ^∗∗^*p* = 0.01, ^∗∗∗^*p* < 0.001). The dashed red line separates adult samples from fledgling samples.

At the sampling unit level, the bacterial phyla with the highest relative abundance of ASVs in fledglings were Proteobacteria [range = 17–45%], Firmicutes [range = 20–34%], Actinobacteria [range = 11–20%], Bacteroidetes [range = 8–20%], Fusobacteria [range = 4–23%], and Synergistetes [range = 3–14%] (Figure 1). Binomial GLM models revealed significant individual variation in the relative abundance of ASVs belonging to a phylum between sampling units (Figure 4). In fledgling samples from the Camargue, the relative abundance of ASVs belonging to a phylum significantly differed between 2013 and 2015 for the Firmicutes and Synergistetes phyla (Figure 4). However, overall differences between fledgling samples from Camargue in 2013 and 2015 were comparatively small relative to differences with other sampling sites (Figure 1, 4). The relative abundance of ASVs belonging to a phylum was significantly different, with large effect sizes, between adult and fledgling samples from the Camargue in 2015 (Figure 1, 4). Adult samples had a much larger relative abundance of ASVs belonging to Fusobacteria [38%] and a much smaller relative abundance of ASVs belonging to Actinobacteria [0.009%] and Synergistetes [0.0001%] compared to fledgling samples (Figure 1, 4).

### Genus Composition Within Phyla

**FIGURE 5:**
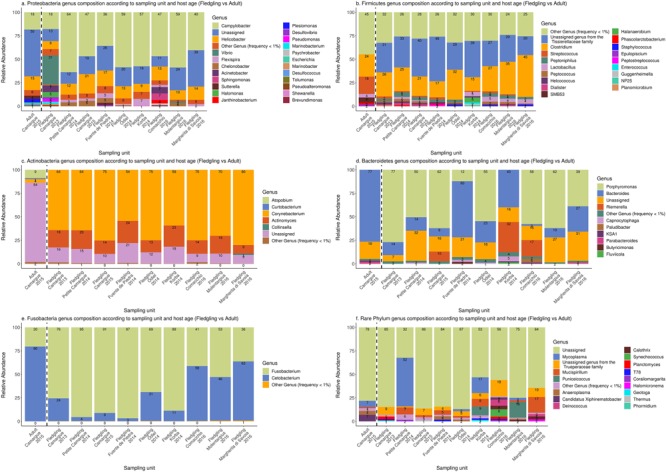
Genus composition with the five most common phyla Proteobacteria **(a)**, Firmicutes **(b)**, Actinobacteria **(c)**, Bacteroidetes **(d)**, and Fusobacteria **(e)** and the other phyla pooled together **(f)**. Only genera with a frequency of at least 1% are represented. Synergistetes was not plotted since it was dominated by a single ASV. Numbers within barplot represent the relative abundance of each genus within the phylum. The dashed black line separates adult samples from fledgling samples.

For fledgling samples, there was strong variation in genus composition within phyla between sampling units, especially among the rarer genus (Figure 5). *Campylobacter* was dominant within Proteobacteria in almost all sampling units [36–59%] except in Camargue in 2013 [18%]. *Helicobacter* was the second most common genus within Proteobacteria in all sampling units [9–22%]. Firmicutes was dominated by an unassigned genus from the *Tissierellaceae* family [20–48%] and *Clostridium* [15–45%]. Actinobacteria was dominated by *Corynebacterium* [54–80%] and to a lesser extent by *Actinomyces* [9–24%]. Within Bacteroidetes, *Porphyromonas* was dominant in many sampling units [39–77%] except in Korba [0.61%] and Fuente de Piedra [12%] in 2014. *Bacteroides* was also prominent in many sampling units [2–60%]. Fusobacteria was dominated by *Fusobacterium* [36–97%] and *Cetobacterium* [3–63%] but again there was strong variation in the contribution of both genus to the phylum across sampling units.

The most striking difference between adult and fledgling samples in Camargue 2015 was the absence of the unassigned genus form the *Tissierellaceae* family within Firmicutes and low presence of *Corynebacterium* in adult samples within Actinobacteria [4%]. *Bacteroides* was also much more dominant in adult samples [77%], largely at the expense of *Porphyromonas* within Bacteroidetes (Figure 5).

Additional information on genus composition of phyla, potential pathogens and variation in their prevalence across sampling units is provided in the Supplementary Material and Supplementary Table S5.

## Discussion

Our results show that fledgling cloacal microbiome, as measured by alpha diversity, beta diversity, the relative abundance of ASVs belonging to a phylum and genus composition within phylum, varied significantly among different colonies of the Greater flamingo in the Mediterranean, and across time within colony sites. The spatio-temporal effects were stronger on individual ASV absence/presence than on ASV abundance (i.e., than on core microbiome composition). Spatial effects had a stronger effect than temporal effects, particularly on ASV abundance. Therefore our study supports the heterogeneous hypothesis whereby local environmental conditions rapidly select and differentiate bacterial communities, thus countering the homogenizing effects of high-dispersing host species. In addition, differences in core microbiome between age classes (adult vs. fledgling samples within the same sampling site) suggests that differences in age-specific environmental, life-history and/or physiological factors between fledglings and adults results in differential selection pressure of core enteric microbiome between adults and fledglings, even within the same environment.

### Temporal and Spatial Variation in Cloacal Microbiome Between Breeding Sites

While several studies have previously shown an effect of sampling site on microbiome differentiation (Klomp et al., 2008; Benson et al., 2010; Amato et al., 2013; Lewis et al., 2017), our study demonstrates microbiome differentiation between breeding sites despite the homogenizing effects of high host dispersal rates in panmictic species. Differences in microbiome between breeding sites were not due to differences in age structure between colonies since differences in alpha and beta diversity between breeding sites remained quantitatively equivalent after controlling for differences in fledgling age (using fledgling tarsus length, a good proxy of fledgling age; see Supplementary Material). Furthermore, differences between sites cannot be explained by parental genetics since previous studies have shown that different breeding colonies form a single genetic population at the scale of the Mediterranean (Geraci et al., 2012; Gillingham et al., 2017). One possible source of variation in microbiome both within and between breeding sites, is the varying degree of fledgling fasting due to variation in parental feeding frequency. Indeed prolonged fasting is known to affect individual gut microbiomes (Crawford et al., 2009; Sonoyama et al., 2009; Costello et al., 2010; Keenan et al., 2013), and has been reported in colonially breeding penguins (Dewar et al., 2014). Whilst data from southern Spain suggests that fledglings do experience some moderate fasting and are not fed daily, most greater flamingo fledglings do not seem to experience severe fasting (Amat et al., 2007). Since fasting duration and individual variation in fasting periods in our study system remains poorly understood, interpreting shifts in microbiome composition as a result of fasting would be very speculative.

Evidence that similar local environmental conditions select for similar cloacal bacterial communities comes from the data we collected in 2014. That year we collected samples from four breeding sites across large geographical distances, from a site in southern France in the north to a site in North Africa in the south. However, two sites sampled in Spain in 2014, Fuente de Piedra and Odiel, were much closer to one another (∼198 km). Greater flamingo adults with chicks at the Fuente de Piedra lagoon do not feed locally, but instead feed mainly in the vicinity of the Odiel breeding site, making regular trips (every 2–3 weeks) between feeding and breeding sites (Amat et al., 2005). Therefore, adults breeding at both sites are feeding at the same sites during the breeding period, and are thus likely to feed chicks with a similar diet (Amat et al., 2005), an important predictor of enteric microbiome diversity in many organisms (Muegge et al., 2011). Consistent with the latter, when taking into account ASV abundance, we found that cloacal microbiome community was more similar between the two Spanish breeding sites than between breeding sites at a greater distance. Therefore, similar environmental factors between the two breeding sites (such as similar food, and associated bacteria from parents) may be driving greater similarity in cloacal communities of fledglings relative to other sites. Nonetheless, fledglings (that were still unable to fly at the time of sampling) are known to ingest soil and feed independently at close proximity of the breeding island (Jenkin, 1957; Johnson and Cézilly, 2007) which may drive the observed significant differentiation in bacterial communities between the two breeding sites at close proximity. Indeed, when taking into account the absence/presence of ASV (which is more sensitive to subtle differences between communities, rather than core differences), we found much stronger differences between the two Spanish breeding sites.

We found that spatial effects had a much stronger effect on beta diversity than temporal effects and explained a larger part of the variation (14% and 18% of the variation of the core microbiome and absence/presence of ASV, respectively, relative to 4% and 8% for temporal effects). The latter is further illustrated by comparing cloacal samples collected in France across three breeding seasons (2013, 2014, and 2015). Due to adverse environmental conditions, in 2014, breeding birds abandoned breeding in the Fangassier lagoon in the Camargue (the regular breeding site) early in the breeding season and bred at a different site in Aigues-Mortes, Petite Camargue, about 34 km away. Despite the fact that 2 years separated the sampling events in Camargue in 2013 and 2015, differences in cloacal microbiome beta diversity was significantly smaller than when comparing these two sampling events to Petite Camargue in 2014 (or any other sampling unit). Therefore, the latter suggests that spatial effects were much stronger than temporal effects even when breeding sites were at close proximity. Indeed, when taking into account the more sensitive absence/presence of ASVs, rather than core differences, we found that Petite Camargue in 2014 actually clustered more closely to the 2016 Italian breeding sites Comacchio, Molentargius and Margherita di Savoia.

We are aware that a 100% match with a sequence known to be a pathogen or a zoonose provides limited evidence that an ASV is a pathogen or a zoonose given the short V4 region amplified in this study (and the lack of survival data associated with the ASV in flamingos). Nonetheless, we believe it does give an indication of the variation of potential enteric pathogen/non-commensal bacterial communities across sampling units. Therefore, our study suggests that fledglings encounter heterogeneous bacterial communities, including potential pathogens, across breeding sites and between years (see Supplementary Table S5). In a previous study, we demonstrated an absence of local genetic differentiation at the level of the major histocompatibility complex (MHC) Class II genes (genes coding for molecules central to the adaptive immune system and the recognition bacterial antigens) among fledgling greater flamingos sampled at various breeding sites across the whole of the Mediterranean (Gillingham et al., 2017). We speculated that either bacterial and pathogen communities were homogenous between breeding colonies (thus precluding selection for host immune genes to adapt to local bacterial communities) or that high gene flow does prevent host local adaptation despite heterogeneous bacterial and pathogen communities (Gillingham et al., 2017). Results obtained in the present study suggest, however, that the very high MHC Class II diversity reported by Gillingham et al. (2017) is most likely the result of selection due to heterogeneous bacterial communities encountered by flamingos across space and time, and that high gene flow between breeding sites (Geraci et al., 2012) is preventing host local adaptation.

### Differences in Enteric Microbiome Communities Between Adults and Fledglings

It was not possible to sample cloacal swabs of live adult birds due to ethical constraints. However, we were able to sample cloacal swabs from two freshly deceased birds and 25 adult fecal samples. Sampling methods (cloacal swabs vs. fecal swabs) can result in different microbiomes (Videvall et al., 2018). However, in our study, cloacal swabs had a high level of fecal matter. Furthermore, despite the sample sizes, both types of sampling methods for adults seem to cluster together in the weighted Unifrac PCoA analyses (Supplementary Figure S3) suggesting that core differences in the microbiome between chicks and adults are not driven by the different sampling methods. Unsurprisingly clustering of adult samples across sampling methods was less clear when using the unweighted Unifrac PCoA analyses (Supplementary Figure S4), since absence/presence analyses will be more sensitive to more subtle differences in the microbiome. Therefore, our results suggests that sampling method had a smaller effect on ASV abundance than on ASV absence/presence and we cannot elucidate whether age class had a stronger effect on ASV absence/presence than on ASV abundance. However, given that samples clustered according to age class rather than sampling method when using the weighted Unifrac distance matrix, our results do suggest a stronger effect of age class than spatio-temporal variation on ASV abundance. Therefore, despite variation between sites and across time in fledgling cloacal microbiome, each age class seems to have a specific enteric microbiome community.

Age differences in enteric microbiome are well documented in the literature (e.g., Yatsunenko et al., 2012; Markle et al., 2013; Saraswati and Sitaraman, 2015; Odamaki et al., 2016) including recently in wild birds (Barbosa et al., 2016; Grond et al., 2017; Kohl et al., 2018). However, the mechanisms that drives differences between age classes in wild populations remains poorly understood.

In Arctic shorebirds, Grond et al. (2017) found that chicks are born with an aseptic cloaca and then experience a rapid increase in enteric bacterial diversity within 10 days. In our study, fledglings were 2–4 months at the time of sampling. We found a negative relationship between tarsus length and alpha diversity suggesting selection for fewer bacteria as fledglings age (see Supplementary Material). However, we found weak evidence for an influence of tarsus length on beta diversity on both ASV abundance and ASV absence/presence, suggesting that differences between young fledglings (∼2 months) and older fledglings (∼4 months) was weak in our study (see Supplementary Material). In conjunction with the lack of difference in alpha diversity between fledgling and adult enteric microbiomes within the same site, our results therefore suggests that fledgling enteric microbiome had already been subject to host selection and was relatively stable at the time of sampling.

At the phylum level, the strongest difference between adult and fledgling enteric microbiome was the much higher relative abundance of Fusobacteria at the expense of Actinobacteria, Synergistetes and Bacteroidetes (Figure 1, 4). Within Actinobacteria, the genus *Corynebacterium*, dominant in fledgling samples, was rare in adult samples. Interestingly, a recent study comparing resident and migratory Red-necked stint, *Calidris ruficollis*, has found that *Corynebacterium* was consistently much more abundant in migratory birds than in resident birds (Risely et al., 2017, 2018). In addition, an experimental approach demonstrated that *Corynebacterium* in the gut microbiome is linked with seasonal host fat deposition in brown bears (Sommer et al., 2016). Risely et al. (2018) suggested that high *Corynebacterium* abundance may enable shorebirds to maximize fat deposition during migration. In greater flamingos, post-fledging dispersal is much higher than adult dispersal (Barbraud et al., 2003; Sanz-Aguilar et al., 2012; Gillingham et al., 2013). For instance in the Camargue, >60% of fledglings (variable between years) disperse to a different wintering site in the autumn, with ∼70% of post-fledging dispersers choosing to winter in Tunisia and the remaining ones wintering in Italy and Spain (Barbraud et al., 2003; Gillingham et al., 2013). In contrast, seasonal dispersal of adults is <10% (Barbraud et al., 2003; Gillingham et al., 2013). Such long-distance dispersal is likely to be energetically expensive for fledglings (Barbraud et al., 2003). Indeed, longer distance post-fledging dispersal to North-Africa is associated with higher mortality than at intermediate sites in Italy and Spain (Sanz-Aguilar et al., 2012). Thus, the high abundance of *Corynebacterium* in fledglings may facilitate fat intake at a crucial development stage in flamingos prior to the undertaking of a costly long-distance trip.

Another conspicuous difference between fledgling and adult enteric microbiome is the absence of *Tissierellaceae* in adults (which is dominant within Firmicutes in fledglings). Such an important difference in Firmicutes between fledglings, a phylum known for its role in nutrient uptake and fat deposition (Liao et al., 2015; Li et al., 2016; Zheng et al., 2016), deserves further investigation in future studies. Indeed, future studies should use the long-term dataset of greater flamingos to investigate the link between long-distance dispersal and cloacal microbiome composition of fledglings, with a particular focus on the *Corynebacterium* genus and *Tissierellaceae* family.

We found more variation in the beta diversity of the microbiome between individuals in fledglings than in adults (using both the weighted and unweighted UniFrac distance matrix; see Supplementary Figures S3, S4). The latter is consistent with what is observed in humans, whereby, as individuals age into adulthood, variation in the microbiome decreases (Yatsunenko et al., 2012). Greater variation in fledgling cloacal microbiome relative to adults also indicates greater potential to detect fitness-microbiome composition associations. Indeed, adult survival in greater flamingos is very high (>90% for birds above 3 years of age) (Cézilly et al., 1996; Tavecchia et al., 2001), and mortality in greater flamingos is at its highest in the early years of life (<80% survival for 1 year olds) (Barbraud et al., 2003; Sanz-Aguilar et al., 2012; Gillingham et al., 2013). Therefore, we predict that the composition of the more variable cloacal microbiome of fledglings is more likely to be linked to fitness than the mature microbiome of adults, although we are aware of no study having investigated the link between fitness and enteric microbiome community in a wild population.

## Conclusion

Host dispersal is known to homogenize the genetic structure of host populations and to prevent local adaptation. In contrast, our results suggest that the homogenizing effects of host dispersal leading to apparent genetic panmixia was disrupted by strong local selection at the scale of host microbiome communities. Furthermore, we found marked differences between fledgling and adult enteric microbiome. Thus, the homogenizing effects of horizontal parental transfer of micro-organisms also seems to be disrupted by local selection within the host. Therefore, selection mechanisms that shape the host’s genetic structure cannot be extended to the genetic structure of the enteric microbiome, which has important implications regarding our understanding of both host local adaptation mechanisms and population genetics of the enteric microbiome.

## Ethics Statement

Ringing and sample collection of greater flamingo chicks were authorized through the personal permit (number 405) of Alan Johnson and Arnaud Béchet delivered by the Centre de Recherche sur la Biologie des Populations d’Oiseaux (CRBPO, Muséum national d’histoire naturelle, France). The study protocol was reviewed and approved by the CRBPO. The Consejería de Medio Ambiente of the Junta de Andalucía (Regional Government) authorized the ringing and sampling of flamingo chicks in Spain. In Italy ringing was authorized by the Regional Authorities of Sardinia, Emilia and Apulia, under the project approval by Istituto Superiore Protezione e Ricerca Ambientale (ISPRA). The Directorate General of Forests (DGF, Direction Générale des Forêts, Ministère de l’Agriculture, des Ressources Hydrauliques et de la Pêche) authorized the ringing and sampling of flamingo chicks in Tunisia through the permit number 2954 issued on August 4, 2014.

## Data Accessibility

The individual gut bacterial 16S rRNA gene raw sequences are available in the NCBI Sequence Read Archive (SRA; accession number: SAMN10576470-SAMN10577345) under BioProject PRJNA485732.

## Author Contributions

MG instigated the study, acquired the funding, collected the samples during field work, did the lab work, analyzed the data, and wrote the first draft of the manuscript. AB participated in acquiring the funding, organized the field work in France across three breeding seasons, and contributed to the writing of the manuscript. FC participated in acquiring the funding and contributed to the writing of the manuscript. KW participated in the field work in France and contributed to the lab work. MR-M, FB, SN, NB, HA, and YK organized the field work in Spain, Italy, and Tunisia and helped to collect the samples. SM participated in the field work in France and Spain and assisted in the data analysis. SS co-instigated the study with MG, participated in acquiring the funding, participated in the field work in France, heads the lab where the work was carried out, and participated in the writing of the manuscript.

## Conflict of Interest Statement

The authors declare that the research was conducted in the absence of any commercial or financial relationships that could be construed as a potential conflict of interest.

## Footnotes

1 https://qiime2.org

## Supplementary Material

FIGURE S1 Genus composition of contamination ASVs isolated from PCR and extraction blanks. Only sequences found with a relative abundance of 1% or more and found in at least three PCR/extraction controls were considered contaminants.

FIGURE S2 **(a)** Odds ratio (exponential of parameter estimates) of the GLM (with a Gamma distribution and log link function) of phylogenetic diversity according to sampling unit and tarsus length. The intercept (dotted black line) is fledgling samples from Camargue in 2015. Odds ratio values are displayed as well as significance relative to the intercept (^∗^*p* = 0.05, ^∗∗^*p* = 0.01, ^∗∗∗^*p* < 0.001). The dashed red line separates the effect of sampling unit from tarsus length. **(b)** Effect of tarsus length on phylogenetic diversity controlling for the effects of sampling unit. Data points are jittered to indicate sample size.

FIGURE S3 Individual data points of weighted UniFrac distance principle coordinates analysis (PCoA) plots of sampling site according sampling site for PCoA axis 1 and 2 **(a)** and PCoA axis 2 and 3 **(b)** and according to age category for PCoA axis 1 and 2 **(c)** and PCoA axis 1 and 3 **(d)**. Ellipses represent 95% confidence intervals.

FIGURE S4 Individual data points of unweighted UniFrac distance principle coordinates analysis (PCoA) plots of sampling site according to sampling site for PCoA axis 1 and 2 **(a)** and PCoA axis 2 and 3 **(b)** and according to age category for PCoA axis 1 and 2 **(c)** and PCoA axis 1 and 3 **(d)**. Ellipses represent 95% confidence intervals.

TABLE S1 Model selection of GAMs (with a Gamma distribution and log link function) of phylogenetic diversity (PD) according to sampling unit and tarsus length. The parameters included in the model are indicated by “+” and degrees of freedom (d.f.), log likelihood (logLik), Akaike Information Criterion for small sample sizes (AICc), delta AICc (Δ AICc), AIC weight (AICc ω), and adjusted-*R*^2^ are shown. Models are ranked according to model support (from the smallest AICc to the largest). The smoother was applied to the parameter sample sequencing depth.

TABLE S2 Model selection of PERMANOVA according to fledgling sampling unit and tarsus length using a dataset with fledgling samples only based on (a) a weighted UniFrac distance matrix and (b) unweighted UniFrac distance matrix. The parameters included in the model are indicated by “+” and degrees of freedom (d.f.), log likelihood (logLik), Akaike Information Criterion for small sample sizes (AICc), delta AICc (Δ AICc), AIC weight (AICc ω), and *R*^2^ are shown. Models are ranked according to model support (from the smallest AICc to the largest).

TABLE S3 Cohen’s *d* and associated 95% confidence intervals for the difference between pairs of centroids following a PCoA analysis using a weighted UniFrac distance matrix (lower diagonal) and unweighted UniFrac distance matrix (upper diagonal). Black units represent the first PCoA axis, red the second and blue the third.

TABLE S4 Model selection of PERMANOVA according to fledgling sampling unit using a dataset with fledgling samples from Camargue only. Models are based (a) on a weighted UniFrac distance matrix and (b) an unweighted Unifrac distance matrix. Analyses were further restricted to samples collected in 2014 only using a (c) weighted UniFrac distance matrix (d) and an unweighted Unifrac distance matrix. The parameters included in the model are indicated by “+” and degrees of freedom (d.f.), log likelihood (logLik), Akaike Information Criterion for small sample sizes (AICc), delta AICc (Δ AICc), AIC weight (AICc ω), and *R*^2^ are shown. Models are ranked according to model support (from the smallest AICc to the largest).

TABLE S5 ASV that were identified as potential pathogens after an NCBI blast search of the sequence.
