# Supplementary figures and images for: Offspring Microbiomes Differ Across Breeding Sites in a Panmictic Species
Source: Front Microbiol. 2019 Feb 6;10:35. doi: 10.3389/fmicb.2019.00035 (PMC6372503; doi:10.3389/fmicb.2019.00035)

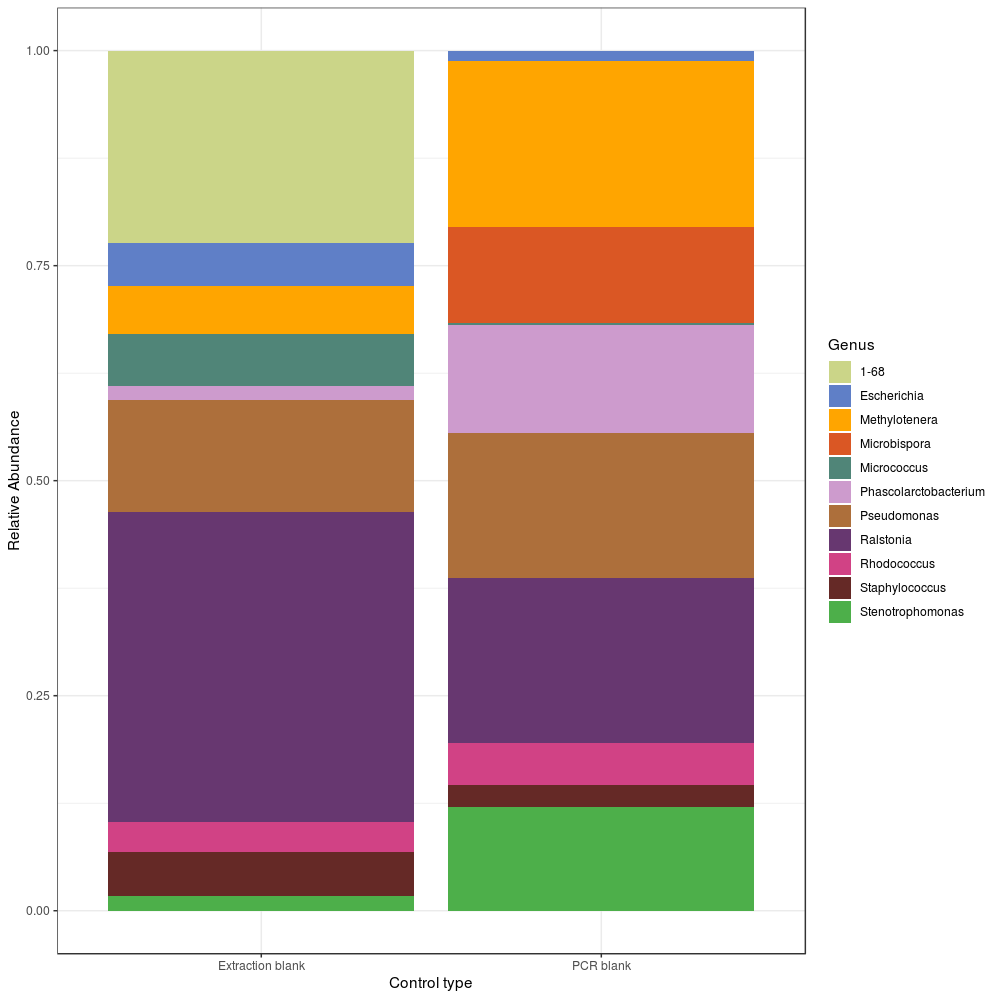

Supplement: FIGURE S1 — Genus composition of contamination ASVs isolated from PCR and extraction blanks. Only sequences found with a relative abundance of 1% or more and found in at least three PCR/extraction controls were considered contaminants. [file Image_1.TIFF]

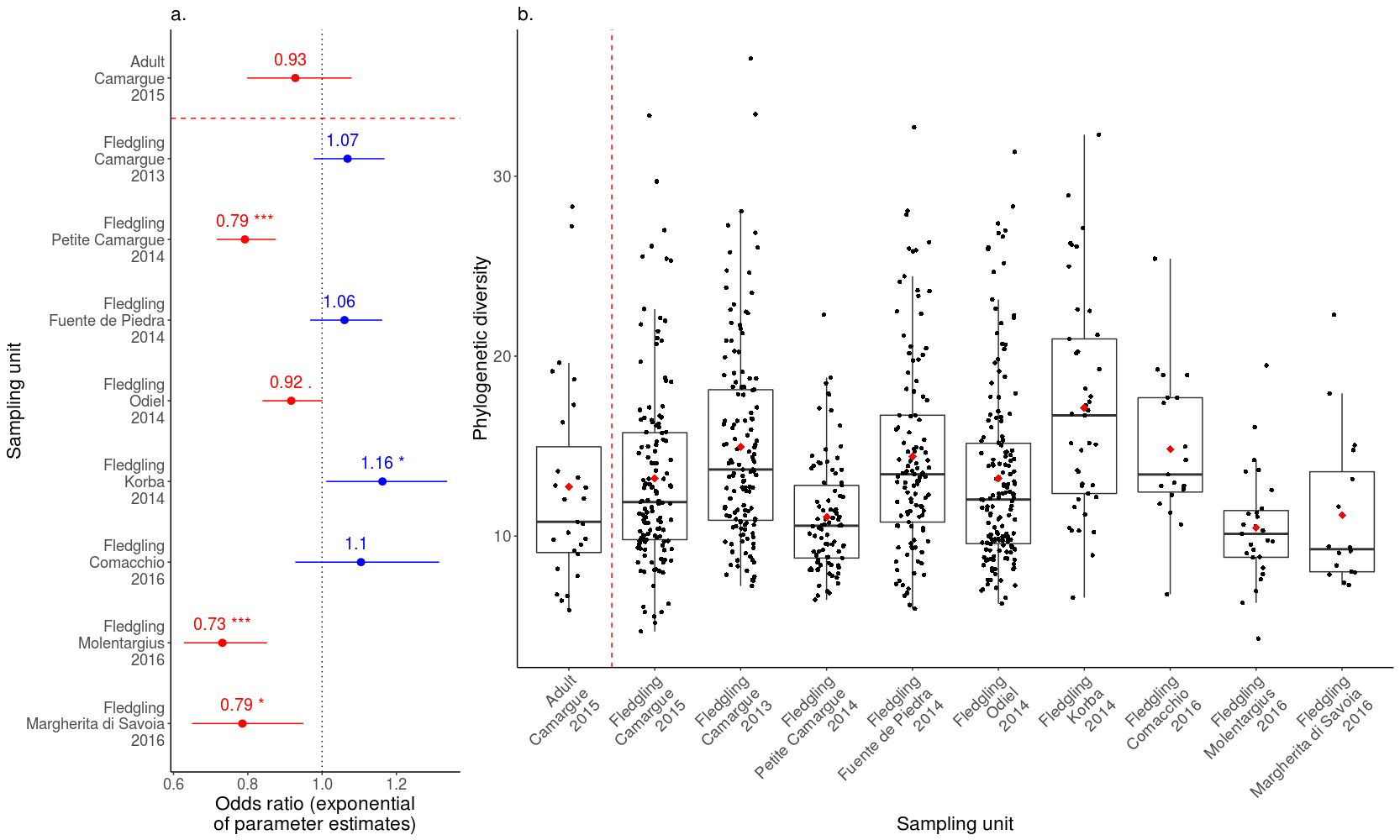

Supplement: FIGURE S2 — (a) Odds ratio (exponential of parameter estimates) of the GLM (with a Gamma distribution and log link function) of phylogenetic diversity according to sampling unit and tarsus length. The intercept (dotted black line) is fledgling samples from Camargue in 2015. Odds ratio values are displayed as well as significance relative to the intercept (∗p = 0.05, ∗∗p = 0.01, ∗∗∗p < 0.001). The dashed red line separates the effect of sampling unit from tarsus length. (b) Effect of tarsus length on phylogenetic diversity controlling for the effects of sampling unit. Data points are jittered to indicate sample size. [file Image_2.TIFF]

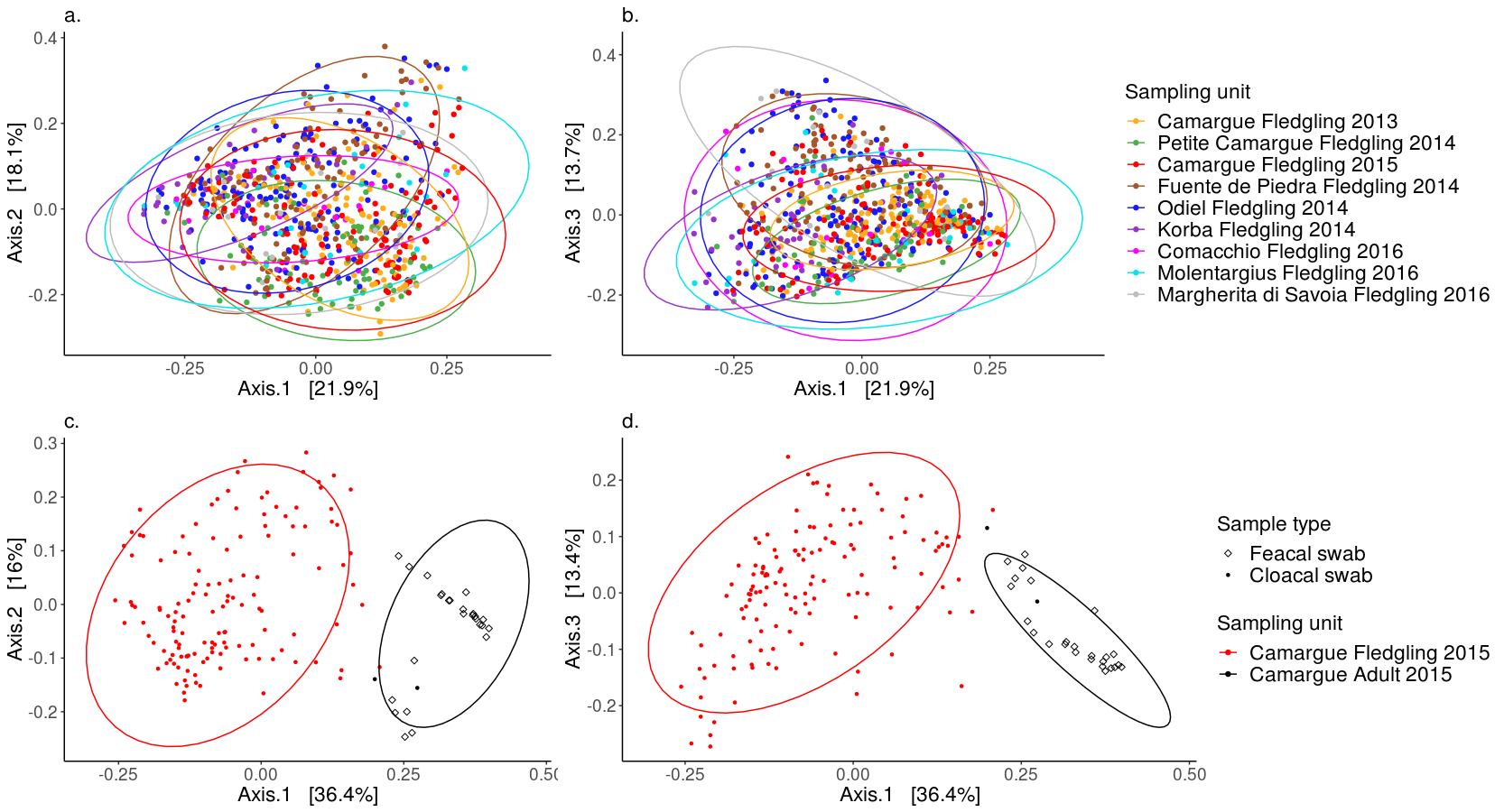

Supplement: FIGURE S3 — Individual data points of weighted UniFrac distance principle coordinates analysis (PCoA) plots of sampling site according sampling site for PCoA axis 1 and 2 (a) and PCoA axis 2 and 3 (b) and according to age category for PCoA axis 1 and 2 (c) and PCoA axis 1 and 3 (d). Ellipses represent 95% confidence intervals. [file Image_3.TIFF]

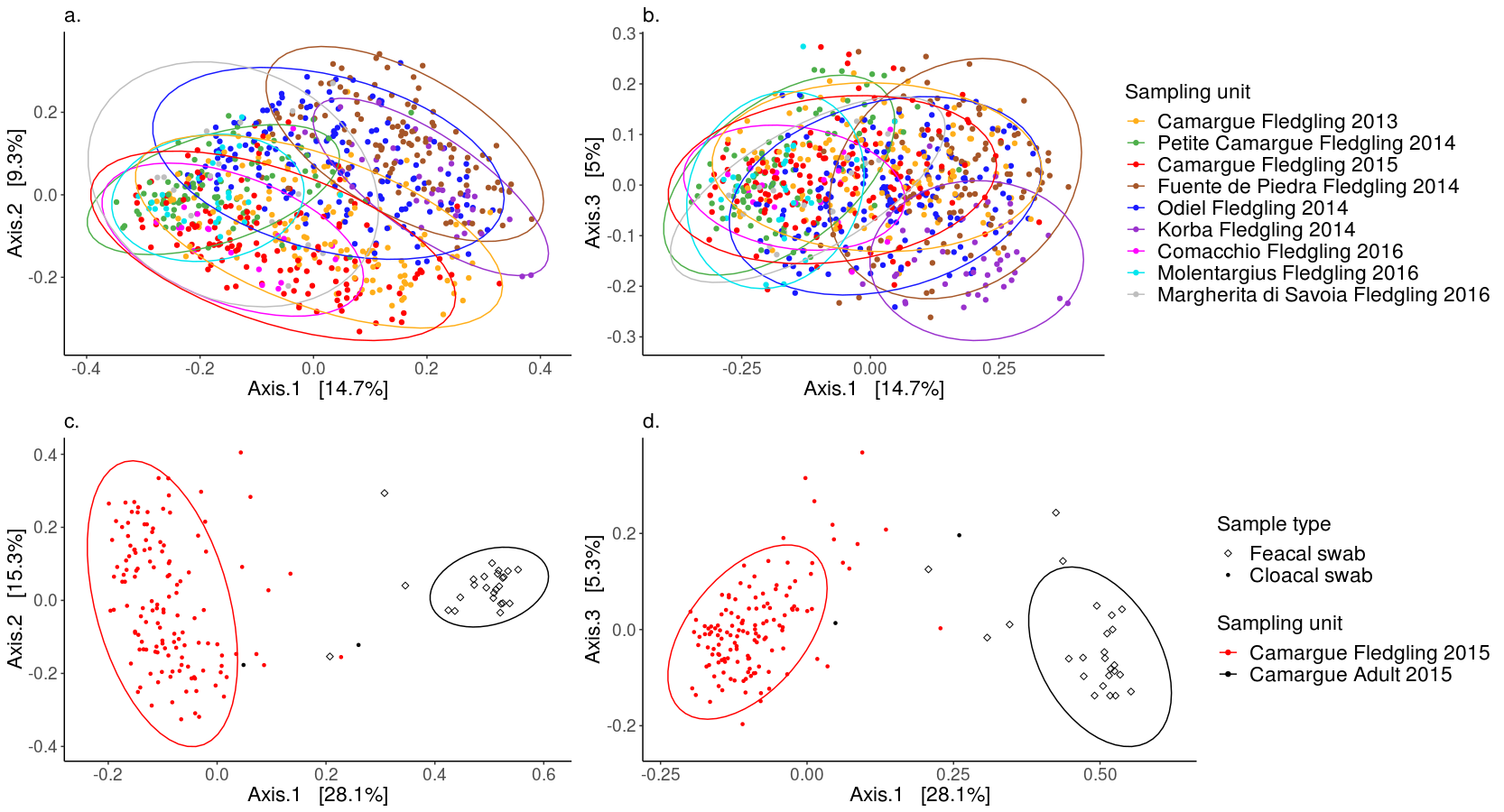

Supplement: FIGURE S4 — Individual data points of unweighted UniFrac distance principle coordinates analysis (PCoA) plots of sampling site according to sampling site for PCoA axis 1 and 2 (a) and PCoA axis 2 and 3 (b) and according to age category for PCoA axis 1 and 2 (c) and PCoA axis 1 and 3 (d). Ellipses represent 95% confidence intervals. [file Image_4.TIFF]
