# Supplementary material for: Offspring Microbiomes Differ Across Breeding Sites in a Panmictic Species
Source: Front Microbiol. 2019 Feb 6;10:35. doi: 10.3389/fmicb.2019.00035 (PMC6372503; doi:10.3389/fmicb.2019.00035)
Supplement: TABLE S1 — Model selection of GAMs (with a Gamma distribution and log link function) of phylogenetic diversity (PD) according to sampling unit and tarsus length. The parameters included in the model are indicated by “+” and degrees of freedom (d.f.), log likelihood (logLik), Akaike Information Criterion for small sample sizes (AICc), delta AICc (Δ AICc), AIC weight (AICc ω), and adjusted-R2 are shown. Models are ranked according to model support (from the smallest AICc to the largest). The smoother was applied to the parameter sample sequencing depth. [file Data_Sheet_1.PDF]

# Supplementary material

## Table of Contents

Bacterial composition of extraction blanks and PCR blanks....2
Controlling for the potential effects of age of fledglings across sampling units....2
Methods....2
Results....2
Table S1: Model selection of GAMs (with a Gamma distribution and log link function) of phylogenetic diversity (PD) according to sampling unit and tarsus length....3
Table S2: Model selection of PERMANOVA according to fledgling sampling unit and tarsus length using a dataset with fledgling samples only based on (a.) a weighted UniFrac distance matrix and (b.) unweighted UniFrac distance matrix....3
Effect size of sampling unit on beta diversity....4
Table S3: Cohen’s D and associated 95% confidence intervals for the difference between pairs of centroids following a PCoA analysis using a weighted UniFrac distance matrix (lower diagonal) and unweighted UniFrac distance matrix (upper diagonal)....4
Disassociating temporal and spatial effects on beta diversity....5
Table S4: Model selection of PERMANOVA according to fledgling sampling unit using a dataset with fledgling samples from Camargue only....5
Detailed description of bacterial phyla and genus, as well as identification of potential pathogens....6
Methods....6
Results....6
Discussion....9
Table S5: ASV that were identified as potential pathogens after an NCBI blast search of the sequence....13
References....15

**Bacterial composition of extraction blanks and PCR blanks**

Removing sequences that were found with a relative abundance of least 1% accross PCR blanks resulted in the removal of 15 ASVs (Figure S1).

**Controlling for the potential effects of age of fledglings across sampling units**

**Methods**

Variation in microbiome between breeding sites may be explained by variation in fledgling age between breeding sites. To control for the latter we repeated analyses on alpha and beta diversity controlling for tarsus length, a very good proxy of fledgling age in tall, lengthy greater flamingos. Of the 731 fledgling samples, we had morphometric data for 697 individuals. All models testing for sampling unit effect on alpha and beta diversity described in the article were repeated using the smaller dataset of 697 individuals but with tarsus length added as an explanatory variable. Model selection was achieved as described in the main article.

**Results**

There was strong support for a negative association between tarsus length and PD (Table S2; Figure S2). However the effect was small (Figure S2) and explained less than 1% of the variation (Table S2). Furthermore, controlling for tarsus length did not change quantitatively the effect of sampling unit since odds ratio are equivalent between both analyses not controlling for tarsus length (Figure 2) and controlling for tarsus length (Figure S2), except for Korba 2014 which was no longer significantly different from Camargue 2015. There was low support of an effect of tarsus length on beta diversity when using both the weighted and unweighted UniFrac distance matrix (Table S2 and S3).

**Table S1: Model selection of GAMs (with a Gamma distribution and log link function) of phylogenetic diversity (PD) according to sampling unit and tarsus length. The parameters included in the model are indicated by "+" and degrees of freedom (d.f.), log likelihood (logLik), Akaike Information Criterion for small sample sizes (AICc), delta AICc (Δ AICc), AIC weight (AICc ω) and adjusted-$R^2$ are shown. Models are ranked according to model support (from the smallest AICc to the largest). The smoother was applied to the parameter sample sequencing depth.**

| Model Rank | Sampling depth | Fledgling Sampling Unit | Tarsus | d.f. | logLik | AICc | Δ AICc | AICc ω | $R^2$ |
|---|---|---|---|---|---|---|---|---|---|
| 1 | + | + | + | 13 | -1996.456 | 4020.2 | 0.00 | 0.808 | 0.153 |
| 2 | + | + | | 12 | -1998.931 | 4023.1 | 2.87 | 0.192 | 0.147 |
| 3 | | + | + | 10 | -2021.978 | 4064.3 | 44.07 | <0.001 | 0.088 |
| 4 | | + | | 9 | -2023.888 | 4066.0 | 45.83 | <0.001 | 0.083 |
| 5 | + | | + | 5 | -2034.584 | 4081.0 | 60.79 | <0.001 | 0.054 |
| 6 | + | | | 4 | -2037.283 | 4084.2 | 64.04 | <0.001 | 0.047 |
| 7 | | | + | 3 | -2051.506 | 4109.0 | 88.84 | <0.001 | 0.007 |
| 8 | | | | 2 | -2054.001 | 4112.0 | 91.81 | <0.001 | 0.000 |

**Table S2: Model selection of PERMANOVA according to fledgling sampling unit and tarsus length using a dataset with fledgling samples only based on (a.) a weighted UniFrac distance matrix and (b.) unweighted UniFrac distance matrix. The parameters included in the model are indicated by "+" and degrees of freedom (d.f.), log likelihood (logLik), Akaike Information Criterion for small sample sizes (AICc), delta AICc (Δ AICc), AIC weight (AICc ω) and R2 are shown. Models are ranked according to model support (from the smallest AICc to the largest).**

| Model Rank | Fledgling Sampling Unit | Tarsus | d.f. | logLik | AICc | Δ AICc | AICc ω | $R^2$ |
|---|---|---|---|---|---|---|---|---|
| | a. weighted UniFrac distance matrix | | | | | | | |
| 1 | + | + | 10 | -63.797 | 147.9 | 0 | 0.540 | 0.14 |
| 2 | + | | 9 | -64.985 | 148.2 | 0.32 | 0.460 | 0.14 |
| 3 | | + | 2 | -115.198 | 234.4 | 86.50 | <0.001 | 0.004 |
| 4 | | | 1 | -116.758 | 237.5 | 89.61 | <0.001 | 0 |
| | b. unweighted UniFrac distance matrix | | | | | | | |
| 1 | + | + | 10 | -329.154 | 678.6 | 0 | 0.658 | 0.19 |
| 2 | + | | 9 | -330.840 | 679.9 | 1.31 | 0.342 | 0.19 |
| 3 | | + | 2 | -399.059 | 802.1 | 123.51 | <0.001 | 0.01 |
| 4 | | | 1 | -402.446 | 808.9 | 130.28 | <0.001 | 0 |

## Effect size of sampling unit on beta diversity

**Table S3: Cohen's D and associated 95% confidence intervals for the difference between pairs of centroids following a PCoA analysis using a weighted UniFrac distance matrix (lower diagonal) and unweighted UniFrac distance matrix (upper diagonal). Black units represent the first PCoA axis, red the second and blue the third.**

| | Camargue Fledgling 2013 | Petite Camargue Fledgling 2014 | Camargue Fledgling 2015 | Fuente de Piedra Fledgling 2014 | Odiel Fledgling 2014 | Korba Fledgling 2014 | Commacchio Fledgling 2016 | Molentargius Fledgling 2016 | Magherita di Savoia Fledgling 2016 | Adult Camargue Fledgling 2015 |
|---|---|---|---|---|---|---|---|---|---|---|
| Camargue Fledgling 2013 | | 1.275<br>[0.985; 1.565]<br>-1.219<br>[-1.507; -0.932]<br>0.110<br>[-0.154; 0.375] | 0.529<br>[0.284; 0.773]<br>0.194<br>[-0.047; 0.435]<br>0.467<br>[0.224; 0.711] | -1.290<br>[-1.566; -1.015]<br>-1.666<br>[-1.957; -1.375]<br>-0.115<br>[-0.366; 0.136] | -0.251<br>[-0.483; -0.020]<br>-1.422<br>[-1.680; -1.163]<br>0.453<br>[0.220; 0.687] | -1.551<br>[-1.892; -1.210]<br>-0.946<br>[-1.261; -0.631]<br>2.082<br>[1.711; 2.453] | 1.024<br>[0.684; 1.365]<br>-0.108<br>[-0.429; 0.212]<br>0.935<br>[0.598; 1.273] | 1.363<br>[1.017; 1.710]<br>-0.997<br>[-1.328; -0.667]<br>0.640<br>[0.320; 0.960] | 0.895<br>[0.556; 1.234]<br>-1.168<br>[-1.518; -0.819]<br>0.664<br>[0.332; 0.996] | -0.889<br>[-1.215; -0.562]<br>-3.477<br>[-3.971; -2.983]<br>2.089<br>[1.701; 2.477] |
| Petite Camargue Fledgling 2014 | 0.033<br>[-0.232; 0.297]<br>-0.840<br>[-1.116; -0.565]<br>0.795<br>[0.521; 1.070] | | -0.699<br>[-0.970; -0.428]<br>1.416<br>[1.121; 1.710]<br>0.336<br>[0.071; 0.601] | -3.037<br>[-3.433; -2.630]<br>-0.708<br>[-0.994; -0.422]<br>-0.200<br>[-0.478; 0.078] | -1.588<br>[-1.875; -1.300]<br>-0.403<br>[-0.656; -0.150]<br>0.329<br>[0.076; 0.581] | -3.562<br>[-4.120; -3.005]<br>0.171<br>[-0.176; 0.518]<br>1.827<br>[1.414; 2.239] | -0.234<br>[-0.616; 0.148]<br>1.382<br>[0.958; 1.806]<br>0.750<br>[0.356; 1.144] | 0.163<br>[-0.205; 0.531]<br>0.164<br>[-0.203; 0.532]<br>0.483<br>[0.111; 0.856] | -0.379<br>[-0.769; 0.010]<br>-0.174<br>[-0.561; 0.213]<br>0.500<br>[0.107; 0.892] | -2.756<br>[-3.269; -2.243]<br>-3.424<br>[-4.000; -2.847]<br>1.790<br>[1.356; 2.225] |
| Grande Camargue Fledgling 2015 | -0.062<br>[-0.302; 0.179]<br>-0.141<br>[-0.381; 0.100]<br>0.261<br>[0.020; 0.503] | -0.088<br>[-0.351; 0.175]<br>0.718<br>[0.446; 0.989]<br>-0.462<br>[-0.728; -0.195] | | -1.880<br>[-2.180; -1.580]<br>-1.837<br>[-2.135; -1.539]<br>-0.513<br>[-0.767; -0.259] | -0.795<br>[-1.034; -0.557]<br>-1.600<br>[-1.864; -1.336]<br>0.011<br>[-0.219; 0.241] | -2.124<br>[-2.496; -1.752]<br>-1.123<br>[-1.443; -0.804]<br>1.599<br>[1.257; 1.940] | 0.479<br>[0.157; 0.802]<br>-0.301<br>[-0.620; 0.019]<br>0.455<br>[0.133; 0.777] | 0.806<br>[0.483; 1.128]<br>-1.176<br>[-1.512; -0.840]<br>0.187<br>[-0.124; 0.498] | 0.356<br>[0.032; 0.680]<br>-1.333<br>[-1.688; -0.978]<br>0.192<br>[-0.130; 0.514] | -1.453<br>[-1.801; -1.105]<br>-3.601<br>[-4.103; -3.100]<br>1.684<br>[1.323; 2.044] |
| Fuente de Piedra Fledgling 2014 | 0.687<br>[0.428; 0.945]<br>0.542<br>[0.287; 0.798]<br>-0.790<br>[-1.051; -0.530] | 0.635<br>[0.350; 0.919]<br>1.337<br>[1.030; 1.644]<br>-1.400<br>[-1.709; -1.091] | 0.666<br>[0.410; 0.923]<br>0.686<br>[0.429; 0.943]<br>-0.943<br>[-1.207; -0.680] | | 1.045<br>[0.790; 1.300]<br>0.260<br>[0.020; 0.500]<br>0.506<br>[0.264; 0.749] | -0.383<br>[-0.704; -0.062]<br>0.743<br>[0.415; 1.072]<br>1.737<br>[1.363; 2.110] | 2.867<br>[2.377; 3.356]<br>1.559<br>[1.167; 1.952]<br>0.814<br>[0.456; 1.171] | 3.349<br>[2.832; 3.866]<br>0.747<br>[0.402; 1.092]<br>0.602<br>[0.261; 0.943] | 2.675<br>[2.197; 3.154]<br>0.467<br>[0.114; 0.819]<br>0.606<br>[0.251; 0.962] | 0.446<br>[0.109; 0.784]<br>-1.688<br>[-2.077; -1.300]<br>1.777<br>[1.383; 2.171] |
| Odiel Fledgling 2014 | 0.749<br>[0.511; 0.988]<br>0.539<br>[0.304; 0.774]<br>-0.051<br>[-0.282; 0.180] | 0.698<br>[0.440; 0.956]<br>1.443<br>[1.162; 1.724]<br>-0.578<br>[-0.834; -0.323] | 0.736<br>[0.499; 0.974]<br>0.696<br>[0.459; 0.933]<br>-0.235<br>[-0.465; -0.004] | 0.076<br>[-0.164; 0.315]<br>-0.055<br>[-0.294; 0.184]<br>0.511<br>[0.268; 0.754] | | -1.337<br>[-1.646; -1.028]<br>0.490<br>[0.207; 0.774]<br>1.459<br>[1.144; 1.773] | 1.337<br>[1.009; 1.664]<br>1.324<br>[0.997; 1.651]<br>0.403<br>[0.104; 0.703] | 1.692<br>[1.354; 2.029]<br>0.483<br>[0.189; 0.777]<br>0.163<br>[-0.127; 0.453] | 1.200<br>[0.875; 1.525]<br>0.222<br>[-0.077; 0.522]<br>0.164<br>[-0.135; 0.464] | -0.655<br>[-0.953; -0.358]<br>-1.979<br>[-2.333; -1.626]<br>1.591<br>[1.259; 1.924] |
| Korba Fledgling 2014 | 1.423<br>[1.088; 1.758]<br>0.821<br>[0.510; 1.132]<br>0.882<br>[0.569; 1.195] | 1.335<br>[0.952; 1.718]<br>2.025<br>[1.598; 2.451]<br>0.116<br>[-0.231; 0.463] | 1.251<br>[0.926; 1.576]<br>1.011<br>[0.696; 1.327]<br>0.545<br>[0.242; 0.847] | 0.633<br>[0.307; 0.959]<br>0.179<br>[-0.140; 0.497]<br>1.394<br>[1.039; 1.748] | 0.545<br>[0.261; 0.830]<br>0.270<br>[-0.011; 0.550]<br>0.618<br>[0.333; 0.904] | | 3.758<br>[2.902; 4.615]<br>0.880<br>[0.340; 1.419]<br>-1.296<br>[-1.862; -0.729] | 4.451<br>[3.551; 5.351]<br>-0.023<br>[-0.506; 0.459]<br>-1.343<br>[-1.878; -0.809] | 3.463<br>[2.627; 4.299]<br>-0.251<br>[-0.782; 0.280]<br>-1.522<br>[-2.122; -0.921] | 1.007<br>[0.494; 1.519]<br>-2.736<br>[-3.407; -2.064]<br>0.292<br>[-0.193; 0.778] |
| Commacchio Fledgling 2016 | 0.703<br>[0.373; 1.033]<br>0.146<br>[-0.175; 0.466]<br>-0.198<br>[-0.123; 0.519] | 0.637<br>[0.246; 1.027]<br>1.236<br>[0.820; 1.651]<br>-0.487<br>[-0.874; -0.101] | 0.635<br>[0.309; 0.961]<br>0.305<br>[-0.015; 0.624]<br>-0.056<br>[-0.374; 0.262] | -0.003<br>[-0.347; 0.340]<br>-0.405<br>[-0.753; -0.058]<br>0.819<br>[0.461; 1.177] | -0.077<br>[-0.374; 0.219]<br>-0.419<br>[-0.719; -0.120]<br>0.164<br>[-0.133; 0.461] | -0.611<br>[-1.138; -0.084]<br>-0.934<br>[-1.476; -0.392]<br>-0.489<br>[-1.011; 0.034] | | 0.477<br>[-0.122; 1.077]<br>-1.056<br>[-1.688; -0.425]<br>-0.242<br>[-0.835; 0.351] | 0.162<br>[-0.522; 0.846]<br>-1.026<br>[-1.753; -0.300]<br>-0.300<br>[-0.386; 0.987] | -3.035<br>[-3.902; -2.167]<br>-3.956<br>[-4.972; -2.939]<br>1.049<br>[0.418; 1.679] |
| Molentargius Fledgling 2016 | 0.247<br>[-0.066; 0.560]<br>0.429<br>[0.114; 0.745]<br>0.514<br>[0.197; 0.831] | 0.203<br>[-0.165; 0.571]<br>1.469<br>[1.056; 1.883]<br>-0.207<br>[-0.575; 0.162] | 0.263<br>[-0.049; 0.574]<br>0.594<br>[0.278; 0.911]<br>0.219<br>[-0.091; 0.530] | -0.371<br>[-0.707; -0.034]<br>-0.143<br>[-0.477; 0.192]<br>1.075<br>[0.718; 1.432] | -0.442<br>[-0.735; -0.148]<br>-0.108<br>[-0.398; 0.182]<br>0.368<br>[0.076; 0.660] | -0.898<br>[-1.404; -0.391]<br>-0.426<br>[-0.914; 0.062]<br>-0.265<br>[-0.750; 0.219] | 0.309<br>[-0.286; 0.904]<br>-0.360<br>[-0.956; 0.236]<br>-0.212<br>[-0.805; 0.381] | | 0.634<br>[0.006; 1.262]<br>-0.256<br>[-0.871; 0.359]<br>-0.002<br>[-0.615; 0.610] | -3.773<br>[-4.680; -2.867]<br>-3.248<br>[-4.076; -2.420]<br>1.175<br>[0.586; 1.764] |
| Magherita di Savoia Fledgling 2016 | 0.902<br>[0.562; 1.241]<br>0.038<br>[-0.286; 0.361]<br>-1.248<br>[-1.601; -0.894] | 0.820<br>[0.418; 1.222]<br>1.033<br>[0.622; 1.444]<br>-1.732<br>[-2.185; -1.279] | 0.802<br>[0.468; 1.135]<br>0.186<br>[-0.136; 0.508]<br>-1.267<br>[-1.619; -0.915] | 0.186<br>[-0.163; 0.534]<br>-0.484<br>[-0.837; -0.131]<br>-0.315<br>[-0.665; 0.035] | 0.112<br>[-0.188; 0.411]<br>-0.515<br>[-0.819; -0.212]<br>-0.697<br>[-1.005; -0.389] | -0.400<br>[-0.934; 0.134]<br>-0.961<br>[-1.520; -0.403]<br>-1.497<br>[-2.095; -0.899] | -0.159<br>[-0.843; 0.525]<br>0.150<br>[-0.534; 0.834]<br>0.930<br>[0.211; 1.649] | -0.438<br>[-1.058; 0.182]<br>0.339<br>[-0.278; 0.956]<br>1.161<br>[0.499; 1.823] | | -2.717<br>[-3.566; -1.868]<br>-2.268<br>[-3.053; -1.483]<br>1.174<br>[0.511; 1.837] |
| Adult Camargue Fledgling 2015 | 2.923<br>[2.475; 3.372]<br>-0.250<br>[-0.563; 0.063]<br>-3.043<br>[-3.501; -2.584] | 2.844<br>[2.323; 3.365]<br>0.654<br>[0.277; 1.031]<br>-3.365<br>[-3.936; -2.795] | 2.400<br>[1.994; 2.807]<br>-0.116<br>[-0.467; 0.194]<br>-2.721<br>[-3.151; -2.291] | 1.868<br>[1.468; 2.268]<br>-0.742<br>[-1.087; -0.397]<br>-1.648<br>[-2.034; -1.262] | 1.708<br>[1.370; 2.047]<br>-0.812<br>[-1.114; -0.511]<br>-1.666<br>[-2.002; -1.330] | 1.349<br>[0.815; 1.884]<br>-1.271<br>[-1.801; -0.742]<br>-2.991<br>[-3.694; -2.289] | 2.139<br>[1.397; 2.880]<br>-0.508<br>[-1.108; 0.093]<br>-1.429<br>[-2.115; -0.743] | 2.098<br>[1.421; 2.775]<br>-0.734<br>[-1.296; -0.173]<br>-2.654<br>[-1.908; -3.400] | 1.738<br>[1.019; 2.457]<br>-0.326<br>[-0.943; 0.290]<br>-2.478<br>[-1.692; -3.264] | |

## Disassociating temporal and spatial effects on beta diversity

**Table S4: Model selection of PERMANOVA according to fledgling sampling unit using a dataset with fledgling samples from Camargue only. Models are based (a.) on a weighted UniFrac distance matrix and (b.) an unweighted Unifrac distance matrix. Analyses were further restricted to samples collected in 2014 only using a (c) weighted UniFrac distance matrix (d) and an unweighted Unifrac distance matrix. The parameters included in the model are indicated by "+" and degrees of freedom (d.f.), log likelihood (logLik), Akaike Information Criterion for small sample sizes (AICc), delta AICc (Δ AICc), AIC weight (AICc ω) and R2 are shown. Models are ranked according to model support (from the smallest AICc to the largest).**

| Model Rank | Fledgling Sampling Unit | df | logLik | AICc | Δ AICc | AICc ω | $R^2$ |
|---|---|---|---|---|---|---|---|
| | a. Dataset with fledgling samples from Camargue only; weighted UniFrac | | | | | | |
| 1 | + | 2 | -16.563 | 45.17 | 0.00 | 0.989 | 0.04 |
| 2 | | 1 | -22.054 | 52.12 | 8.95 | 0.011 | 0.00 |
| | b. Dataset with fledgling samples from Camargue only; unweighted UniFrac | | | | | | |
| 1 | + | 2 | -117.077 | 246.20 | 0.00 | 1.000 | 0.08 |
| 2 | | 1 | -128.698 | 265.41 | 21.21 | <0.001 | 0.00 |
| | c. Dataset with fledgling samples from 2014; weighted UniFrac | | | | | | |
| 1 | + | 4 | -43.020 | 106.14 | 0.00 | 1.000 | 0.14 |
| 2 | | 1 | -73.489 | 154.99 | 54.85 | <0.001 | 0.00 |
| | d. Dataset with fledgling samples from 2014; unweighted UniFrac | | | | | | |
| 1 | + | 4 | -193.00 | 406.10 | 0.00 | 1.000 | 0.18 |
| 2 | | 1 | -232.86 | 473.73 | 73.63 | <0.001 | 0.00 |

**Detailed description of bacterial phyla and genus, as well as identification of potential pathogens**

**Methods**

We investigated if ASV were potentially pathogenic by blasting ASV sequence using the NCBI database. We reported any ASV with a 100% match with a sequence known to be a pathogen or a zoonose in birds and estimated it's prevalence across sampling units. We are aware that a 100% match with a sequence known to be a pathogen or a zoonose is not proof that the ASV is a pathogen or a zoonose given the short V4 region amplified in this study (and the lack of survival data associated with the ASV in flamingos). Nonetheless, we believe it does give an indication of the variation of potential enteric pathogen/non-commensal bacterial communities across sampling units.

**Results**

*Campylobacter* was the most dominant Proteobacteria genus within most sampling units (Figure 5; 18-59%). Seventy-seven percent of Campolybacter ASVs had a 96-98.8% NCBI blast hit with *Campylobacter canadensis,* with high prevalence in all sites (range: 77.8-95.1%). A *Campylobacter* ASV with a 100% NCBI blast hit for *Campylobacter jejuni* and *Campylobacter coli* was detected but at relatively low prevalence across sampling units and not detected in some sampling units with low sample sizes (Table S5). A *Campylobacter* ASV with a 100% NCBI blast hit for *Campylobacter hepaticus* was detected in all sites except in Comacchio in 2016 with a prevalence range of 11.1-25% (Table S5). Nineteen percent of *Campylobacter* assigned sequences had a NCBI blast hit of 97.1-99.6% for the closely related *Campylobacter jejuni, Campylobacter coli, Campylobacter hepaticus, Campylobacter insulaenigrae* and *Campylobacter helveticus* with a strong variation in prevalence between fledgling sampling units (1.8-81.5%; Table S5). An ASV had a 100% NBCI hit with *Campylobacter lari* with prevalence ranging 14.8-54% between sampling units (Table S5).

The second most common assigned Proteobacteria genus was *Helicobacter* (Figure 5). Sixty-eight percent of Helicobacter ASVs had a 98-100% NCBI blast hit with Helicobacter sp. strain 91-266-11 (NCBI Accession number: M88152) previously isolated in the feces of ferrets (Fox et al., 1992), with prevalence ranging 63-81.3% between sampling units. Twenty-five percent of *Helicobacter* ASV had an NCBI hit of 98.4-99.2% with *Helicobacter pullorum* and *Helicobacter pametensis,* with prevalence ranging 25.9-75.6% between sampling units. A *Helicobacter* ASV with a 100% NCBI blast hit for *Helicobacter hepaticus* was detected in all sites except in Comacchio in 2016 with a prevalence range of 11.1-25%. A *Helicobacter* ASV with a 100% NCBI blast hit for the potential zoonose *Helicobacter valdiviensis* was detected but at relatively low prevalence (0-24.7%) across sampling units (Table S5).

There was a high proportion (29%) of Proteobacteria ASVs assigned to *Vibrio* sps. in samples collected from fledglings in Camargue 2013 (Figure 5). Ninety-two percent of Vibrio assigned ASVs belonged to a single ASV with 100% NCBI blast hit with several *Vibrio* sps. (*Vibrio harveyi*, *Vibrio alginolyticus*, *Vibrio azureus, Vibrio antiquarius,* and *Virbio diabolicus*). Prevalence of the latter ASV was 76.7% for samples collected from fledglings in Camargue in 2013, and 56.1% for samples collected from fledglings in Korba in 2014 but was much lower for all other sites with a range of 0-18.8%.

The high relatedness between *Echerichia* sps., *Salmonella* sps. and *Shigella* sps. meant it was not possible to distinguish between these Enterobacteriaceae using the methods of this study. However their combined prevalence varied between sampling units with a range of 31.3-85% (Table S5; Figure 5). One ASV had a 100% NCBI blast hit with the potentially pathogenic *Pseudomonas aeruginosa*, with a relatively high prevalence in Camargue in 2015 but a very low prevalence across all other sampling units (0-10.5%; Table S5).

Within the Firmicutes phylum, ASVs from the *Tissierellaceae* family dominated fledglings samples but not adult samples (Figure 5). ASVs from the *Clostridium* genus also constituted a high

proportion of sequences from the *Fimicutes* phylum (Figure 5). Twenty-one percent of *Clostridium* sequences assigned to an ASV with a 100% NCBI hit with a sequence isolated in shorebird cloacal 16S microbiome (Ryu et al., 2014). The second most common *Clostridium* ASV (19.5% of *Clostridium* sequences) had a 100% NCBI hit with *Clostridium perfringens* with a prevalence range of 28.1-82.7% across all sampling units.

Actinobacteria was dominated by *Corynebacterium* followed by *Actinomyces* in fledgling sampling units (Figure 5). In contrast, in adult samples collected in Camargue in 2015, *Corynebacterium* was not dominant (Figure 5) and there was a much higher proportion of ASVs assigning to *Atopobium*. There was strong variation in the genus composition of *Bacteroidetes* between sampling units, with *Porphyromononas* dominant in some sampling units but not in others and similarly strong variation in the proportion of *Bacteroides* between sampling units (Figure 5). There was a high proportion of sequences assigned to *Riemerella* in fledgling samples collected in Korba 2014, Comacchio 2016 and Camargue 2015 (Figure 5). Sixty-four percent of *Riemerella* sequences assigned to a ASV with a 100% NCBI hit with the potentially pathogenic *Riemerella anatipestifer*, with a strong variation in prevalence between sampling units (0-57.8%; Table S5). Another ASV had a 100% NCBI hit with the potentially pathogenic *Riemerella columbina* but at a much lower prevalence than *R. anatipestifer* across sampling units (0-19.5%; Table S5). Fusobacteria was dominated by *Fusobacterium* and *Cetobacterium* but again there was strong variation in the contribution of both genus to the phylum across sampling units (Figure 5). 99.5% of ASVs assigned as Synergistetes sequences had an 86-88% NCBI blast hit with *Jonquetella* sp. isolated from vaginal swabs in humans.

Finally, among the rare phylum we identified ASVs that assigned to the class Chlamydiia and 86% of Chlamydiia sequences had a 97.2-98% NCBI hit with the potentially pathogenic *Chlamydophila abortus, Chlamydia psittaci* and *Chlamydia gallinacea*.

**Discussion**

Previous studies have found that Proteobacteria and Firmicutes dominate the gut microbiota in bird species that are carnivorous or with generalists diets, including marine invertebrates (Blanco, 2014; Grond et al., 2014; Hird et al., 2014; Risely et al., 2017, 2018; Ryu et al., 2014). Consistent with the latter Proteobacteria (17-45%) and Firmicutes (20-34%) also represented a large proportion of the cloacal microbiome in greater flamingos. The function of enteric Proteobacteria in wild birds is unknown, although they are known to be more abundant than in mammalian hosts (17-45% in this study, 13.8-33% in shorebireds (Grond et al., 2014; Ryu et al., 2014) compared to < 10% in mammals (Ley, Hamady, et al., 2008)). However, Proteobacteria are also known to include a range of avian pathogens within the genus *Campylobacter, Helicobacter, Echerichia, Salmonella, Shigella,* and *Vibrio* (Benskin et al., 2009; Shi et al., 2014). All of the latter Proteobacteria genus were detected in greater flamingos cloacal microbiome with a relative abundance above 1% in at least one sampling unit (Figure 5), although it was not possible to distinguish between *Echerichia, Salmonella, Shigella* since they have identical sequences within the V4 region amplified in this study.

In most sampling units (except in Camargue in 2013), the *Campylobacter* genus dominated the Proteobacteria phylum and *Helicobacter* was the second most common genus (Figure 5). *Campulabacter spp.* and *Helicobater sps* have been intensively studied in wild birds since they are usually associated with pathogens, including *C. jejuni, C. coli, C. hepaticus, C. lari* and *H. pylori* (Benskin et al., 2009; Collado et al., 2014; Ryu et al., 2014; Waldenström et al., 2002). A large prevalence in cloacal microbiome of *Campylobacter* have previously been reported in shorebirds (Grond, 2017; Grond et al., 2014; Risely et al., 2017) and apparently healthy wild birds across species are known to harbor various *Campylobacter* sps. within the cloacal flora, including potentially pathogenic strains, suggesting that they might be a healthy part of the intestinal flora in some birds (Benskin et al., 2009; Kapperud and Rosef, 1983; Ryu et al., 2014). Indeed, the most

common Campylobacter ASVs in cloacal fledglings of greater flamingos (77% of Campylobacter sequences with a prevalence of 77.8-95.1% across sites) appeared to be closely related to *Campylobacter canadensis* first isolated in whooping cranes in Canada (*Grus americana*) (Inglis et al., 2007). Similarly 68% of Helicobacter ASVs appeared closely related to *Helicobacter sp.* strain 91-266-11 (NCBI Accession number: M88152) previously isolated in the feces of ferrets (Fox et al., 1992), with a prevalence ranging 63-81.3% between sampling units. The high prevalence and abundance of the latter *Campylobacter* and *Helicobacter* ASVs suggests that they are a commensal and/or mutualistic part of fledgling cloacal flora in greater flamingos. Nonetheless the detection of *Campylobacter* and *Helicobacter* ASVs with a 100% NCBI hit with potentially pathogenic species, such as *C. jejuni, C. coli, C. hepaticus, C. lari* and *H. valdiviensis,* at a comparatively low abundance but at varying prevalence across sites does suggest that greater flamingos maybe an important reservoir of *Campylobacter* and *Helicobacter* pathogens in Mediterranean wetlands. Another potentially pathogenic Proteobacteria detected in this study was *Pseudomonous aeruginosa* which has been reported as an opportunistic pathogen during waves of mass mortality of lesser flamingos (*Phoeniconaias minor*) in East Africa (Kock et al., 1999; Krienitz and Kotut, 2010). Furthermore, a mass mortality event of ~30, 000 lesser flamingos in 2006 in Kenya was attributed to a *P. aeruginosa* outbreak (Krienitz and Kotut, 2010). Although the prevalence of the *P. aeruginosa* ASV was low in most sampling units, in Camargue in 2015 the prevalence was 25.2% for fledglings and 7.4% for adults suggesting strong temporal variation for this ASV. However the pathogenicity of the *P. aeruginosa* infection in greater flamingos remains unknown.

Firmicutes are known to be associated with nutrient uptake, the rate of metabolism and weight gain in both mammals and domesticated birds (Li et al., 2016; Liao et al., 2015; Zhang et al., 2014; Zheng et al., 2016). The abundance of Firmicutes was lower in greater flamingos (20-34%) than previously reported in Arctic breeding shorbirds of North America (~55%) (Grond, 2017; Grond et al., 2014) but comparable or higher to the Red-necked stint (*Calidris ruficollis*) (14%)

(Risely et al., 2017). Unassigned genus from the *Tissierellaceae* family and *Clostridium* dominated Firmicutes in fledgling samples. Little is known about *Tissierellaceae* although it has been linked with anaerobic digestion in biogas from slaughterhouse wastewater (Granada et al., 2018). An ASV with a 100% NCBI blast hit with the potential zoonose *Clostridium perfringens* was found at a relatively high prevalence in greater flamingo cloacal microbiome but again with strong variation in prevalence between sampling sites. *Clostridium perfringens* is frequently found in healthy birds but can cause outbreaks of acute clinical disease in poultry flocks and is an important source of food poisoning in humans  (Engström et al., 2003).

Actinobacteria was the third phylum that dominated greater flamingo cloacal microbiome of fledglings (11-20%) (but intriguingly not of adults see main text of the manuscript). There are no studies investigating the role of Actinobacteria in birds but in mammals it is associated with fiber intake (Dominianni et al., 2015), seasonal fat intake (Sommer et al., 2016) and in bees with carbohydrate breakdown (Lee et al., 2015). *Bacteriodetes* in greater flamingos were found at a similar relative abundance to shorebirds (~7-20%) (Grond, 2017; Grond et al., 2014; Risely et al., 2017). *Bacteriodetes* encompass bacteria that have a mostly mutualistic relationship with their host, which are known to play an important role in the breakdown of carbohydrates and plant cell walls in mammals (Thomas et al., 2011).  However, two ASVs from *Bacteriodetes* were found to have a 100% NCBI hit with the potential zoonoses *Riemerella anatipestifer* and *Riemerella columbina*. *Riemerella anatipestifer* is known to cause septicaemia and death in birds, particularly young duck and geese, (Ryll et al., 2001) and *Riemerella columbina* is associated with respiratory disease in pigeons (Vancanneyt et al., 1999).  Prevalence of the ASV matching *Riemerella anatipestifer* was extremely variable reaching 57.8% in fledglings in Camargue in 2015 (and 7.4% in adults in the same site) but completely absent in other sites including Petite Camargue in 2014, Fuente de Piedra in 2014 and Molentargius in 2016. *Riemerella columbina* followed a very similar pattern to *Riemerella anatipestifer* but a lower prevalence.  Although *Riemerella anatipestifer* infection is

commercially important, it has also been suggested to be a part of the healthy pharyngeal flora of ducks (Ryll et al., 2001).

A fifth phylum that dominated the greater flamingo gut microbiota is Fusobacteria (4-23%), which are commonly found in the gut microbiota of birds (Barbosa et al., 2016; Bennett et al., 2013; Dewar et al., 2014; Grond et al., 2014; Hird et al., 2014; Risely et al., 2017, 2018). The high dominance of the *Cetobacterium,* first detected in sea mammals (Foster et al., 1995), and *Fusobacterium* genus within Fusobacteria has also been reported in shore birds (Grond, 2017; Grond et al., 2014; Risely et al., 2017). *Cetobacterium* is commonly isolated in the gut of several fish species (Larsen et al., 2014; Liu et al., 2016; Tsuchiya et al., 2008). However the functional importance of *Cetobacterium* and *Fusobacterium* in wild bird cloacal flora remains unknown. Finally a sixth dominant phylum found in fledgling cloacal microbiome was Synergistetes (3-14%), which was dominated by a single ASV. Synergistetes has only been clearly defined as a phylum in 2009 (Jumas-Bilak et al., 2009) and a member of Synergistetes was first isolated in the rumen of sheep (Allison et al., 1992). To our knowledge this is the first report of Synergistetes in cloacal flora of wild birds and its potential function is completely unknown.

In other non-dominant phylum, we identified *Mycoplasma iowae* as a potential zoonose. *Mycoplasma* is a genus within the *Tenericutes* phylum and *Mycoplasma iowae* has been isolated in gastrointestinal tract of a number of avian hosts and is known to cause embryo mortality in turkey (Al-Ankari and Bradbury, 1996). However *Mycoplasma iowae* was found at a very low prevalence across breeding sites. Several ASVs of the Class *Chlamydiia* were also identified. All known members of *Chlamydiia* only grow by infecting eurkyotic cells (Vanrompay et al., 1995), however all the *Chlamydiia* ASVs strains identified in this study did not have a 100% NCBI similarity with known *Chlamydiia* species.

**Table S5: ASV that were identified as potential pathogens after an NCBI blast search of the sequence.**

| ASV which are potentially pathogenic and/or zoonotic | Description | NCBI hit | Number of unique ASV | Prevalence per sampling unit | | | | | | | | | |
|---|---|---|---|---|---|---|---|---|---|---|---|---|---|
| | | | | Adult Camargue 2015 | Fledging Camargue 2013 | Fledging Petite Camargue 2014 | Fledging Camargue 2015 | Fledging Fuente de Piedra 2014 | Fledging Odiel 2014 | Fledging Korba 2014 | Fledging Comacchio 2016 | Fledging Molentargius 2016 | Fledging Margherita di Savoia 2016 |
| *Campylobacter jejuni; Campylobacter coli;* | *C. jejuni* and and *C. coli* are one of the most common causes of bacterial infections in humans worldwide. Usually commensal in birds. | 100% | 1 | 0% (0/27) | 10.5% (14/133) | 4.5% (4/89) | 16.8% (2/135) | 16.8% (19/113) | 14.6% (23/158) | 4.9% (2/41) | 0% (0/19) | 3.7% (1/27) | 0% (0/16) |
| *Campylobacter hepaticus* | *C. hepaticus* is the cause of spotty liver disease in chickens. | 100% | 2 | 18.5% (5/27) | 15.8% (21/133) | 19.1% (17/89) | 11.1% (15/135) | 16.8% (19/113) | 17.7% (28/158) | 17.1% (7/41) | 0% (0/19) | 14.8% (4/27) | 25% (4/16) |
| *Campylobacter jejuni; Campylobacter coli; Campylobacter hepaticus; Campylobacter insulaenigrae; Campylobacter helveticus* | For *C. jejuni; C. coli and C. hepaticus* see above. *C. insulaenigrae* can cause enteritis and septicaemia in humans. | 97.1-99.6% | 17 | 0% (0/27) | 63.2% (84/133) | 68.5% (61/89) | 80.7% (109/135) | 1.8% (2/113) | 3.2% (5/158) | 78.0% (32/41) | 78.9% (15/19) | 81.5% (22/27) | 56.5% (9/16) |
| *Campylobacter lari* | Commonly found in seagull feces but known to be involved in cases of enteritis, severe abdominal pain and terminal bacteremia in humans | 100% | 1 | 14.8% (4/27) | 22.6% (30/133) | 31.5% (28/89) | 23% (31/135) | 54% (61/113) | 22.8% (36/158) | 46.3% (19/41) | 31.6% (6/19) | 18.5% (5/27) | 25% (4/16) |
| *Campylobacter lari* | Commonly found in seagull feces but know to involved in cases of enteritis, severe abdominal pain and terminal bacteremia in humans | 99.6% | 2 | 0% (0/27) | 0% (0/133) | 3.4% (3/89) | 0% (0/135) | 0% (0/113) | 0% (0/158) | 4.9% (2/41) | 0% (0/19) | 0% (0/27) | 0% (0/16) |
| *Helicobacter valdiviensis* | H. valdiviensis is an avian enterohepatic species that is a potential human intestinal pathogen | 100% | 1 | 0% (0/27) | 3.8% (5/133) | 24.7% (22/89) | 5.9% (8/135) | 16.8% (19/113) | 5.7% (9/158) | 22% (9/41) | 0% (0/19) | 7.4% (2/27) | 0% (0/16) |
| *Helicobacter valdiviensis* | *H. valdiviensis* is an avian enterohepatic species that is a potential human intestinal pathogen | 99.2-99.6% | 4 | 0% (0/27) | 0% (0/133) | 0% (0/89) | 0% (0/135) | 11.5% (13/113) | 0.6% (1/158) | 2.4% (1/41) | 0% (0/19) | 11.1% (3/27) | 6.3% (1/16) |
| *Escherichia sps.; Salmonella sps.; Shigella sps.* | *Echerichia sps.* are known to be part of commensal gut flora, although some strains are know to be important pathogens. *Salmonella* are intracellular pathogens. *Shigella* is an enteropahtogen. | 100% | 12 | 51.9% (14/27) | 73.7% (98/133) | 36% (32/89) | 31.9% (43/135) | 85% (96/113) | 54.4% (86/158) | 65.9% (27/41) | 15.8% (3/19) | 55.6% (15/27) | 31.3% (5/16) |
| *Salmonella sps.; Proteus mirabilis* | *Salmonella* are intracellular pathogens. *P. mirabilis* is a dominant cause of *Proteus* infections in humans. | 100% | 1 | 0% (0/27) | 0% (0/133) | 1.1% (1/89) | 0% (0/135) | 1.8% (2/113) | 0% (0/158) | 39% (16/41) | 0% (0/19) | 0% (0/27) | 0% (0/16) |

| | | | | | | | | | | | | | |
|---|---|---|---|---|---|---|---|---|---|---|---|---|---|
| *Pseudomonous aeruginosa* | *P. aeruginosa* can be an opportunistic pathogen in plants and humans. It has also been reported to infect lesser flamingos during waves of mass mortality. | 100% | 1 | 7.4% (2/27) | 0.8% (1/133) | 0% (0/89) | 25.2% (34/135) | 1.8% (2/113) | 0% (0/158) | 0% (0/41) | 10.5% (2/19) | 0% (0/27) | 0% (0/16) |
| *Clostridium perfringens* | Often found in the intestinal tract of healthy birds but can cause outbreaks of both acute clinical disease and subclinical disease in captive chickens and turkeys. Also a common cause of food poisoning in humans. | 100% | 2 | 37% (10/27) | 82.7% (110/133) | 28.1% (25/89) | 52.6% (71/135) | 63.7% (72/113) | 55.1% (87/158) | 12.2% (5/41) | 73.7% (14/19) | 25.9% (7/27) | 68.8% (11/16) |
| *Clostridium perfringens* | Often found in the intestinal tract of healthy birds but can cause outbreaks of both acute clinical disease and subclinical disease in captive chickens and turkeys. Also a common cause of food poisoning in humans. | 93.7-99.6% | 13 | 37% (10/27) | 4.5% (6/133) | 0% (0/89) | 5.9% (8/135) | 11.5% (13/113) | 7% (11/158) | 0% (0/41) | 10.5% (2/19) | 29.6% (8/27) | 12.5% (2/16) |
| *Riemerella anatipestifer* | *R.anatipestifer* causes septicaemia and death in young duck and geese | 100% | 2 | 7.4% (2/27) | 4.5% (6/133) | 0% (0/89) | 57.8% (78/135) | 0% (0/113) | 5.1% (8/158) | 24.3% (10/41) | 31.6% (6/19) | 0% (0/27) | 18.8% (3/16) |
| *Riemerella anatipestifer* | *R.anatipestifer* causes septicaemia and death in young duck and geese | 98.4-99.2% | 2 | 3.7% (1/27) | 0% (0/133) | 0% (0/89) | 11.1% (15/135) | 0% (0/113) | 1.3% (2/158) | 19.5% (8/41) | 10.5% (2/19) | 0% (0/27) | 0% (0/16) |
| *Riemerella columbina* | Associated with respiratory disease in pigeons | 100% | 1 | 3.7% (1/27) | 0% (0/133) | 0% (0/89) | 11.1% (15/135) | 0% (0/113) | 1.3% (2/158) | 19.5% (8/41) | 10.5% (2/19) | 0% (0/27) | 0% (0/16) |
| *Mycoplasma iowae* | *M. iowae* is an avian mycoplasma which tends to infect the gastrointestinal tract. It can cause chick and embryo mortality in turkey and chicken. | 100% | 1 | 0% (0/27) | 0% (0/133) | 3.4% (3/89) | 0.7% (1/135) | 0% (0/113) | 8.2% (13/158) | 4.9% (2/41) | 0% (0/19) | 7.4% (2/27) | 6.25% (1/16) |
| *Mycoplasma iowae* | *M. iowae* is an avian mycoplasma which tends to infect the gastrointestinal tract. It can cause chick and embryo mortality in turkey and chicken. | 99.6% | 6 | 0% (0/27) | 0% (0/133) | 2.2% (2/89) | 0% (0/135) | 0% (0/113) | 0% (0/158) | 0% (0/41) | 0% (0/19) | 0% (0/27) | 0% (0/16) |
| *Chlamydophila abortus; Chlamydia psittaci; Chlamydia gallinacea* | *C. abortus* causes abortion and fetal death in mammals, including humans. C. psittaci can cause endemic avian chlamydiosis. *C. gallinacea* is known to infect chickens and causes reduction in body weight. | 97.2-98% | 8 | 0% (0/27) | 0% (0/133) | 1.1% (1/89) | 1.5% (2/135) | 0% (0/113) | 11.3% (18/158) | 9.8% (4/41) | 5.3% (1/19) | 3.7% (1/27) | 6.3% (1/16) |

## References


Al-Ankari, A.-R. S., and Bradbury, J. M. (1996). Mycoplasma iowae: A review. *Avian Pathol.* 25, 205–229. doi:10.1080/03079459608419137.

Allison, M. J., Mayberry, W. R., Mcsweeney, C. S., and Stahl, D. A. (1992). Synergistes jonesii, gen. nov., sp.nov.: A Rumen Bacterium That Degrades Toxic Pyridinediols. *Syst. Appl. Microbiol.* 15, 522–529. doi:10.1016/S0723-2020(11)80111-6.

Barbosa, A., Balagué, V., Valera, F., Martínez, A., Benzal, J., Motas, M., et al. (2016). Age-Related Differences in the Gastrointestinal Microbiota of Chinstrap Penguins (Pygoscelis antarctica). *PLOS ONE* 11, e0153215. doi:10.1371/journal.pone.0153215.

Bartón, K. (2016). *MuMIn: Multi-Model Inference. R package version 1.15.6.* Available at: https://CRAN.R-project.org/package=MuMIn.

Battaglia, T. (2018). *btools: A suite of R function for all types of microbial diversity analyses*.

Bennett, D. C., Tun, H. M., Kim, J. E., Leung, F. C., and Cheng, K. M. (2013). Characterization of cecal microbiota of the emu (Dromaius novaehollandiae). *Vet. Microbiol.* 166, 304–310. doi:10.1016/j.vetmic.2013.05.018.

Benskin, C. M. H., Wilson, K., Jones, K., and Hartley, I. R. (2009). Bacterial pathogens in wild birds: a review of the frequency and effects of infection. *Biol. Rev.* 84, 349–373. doi:10.1111/j.1469-185X.2008.00076.x.

Blanco, G. (2014). Influence of diet on the gastrointestinal flora of wintering red kites. *Eur. J. Wildl. Res.* 60, 695–698. doi:10.1007/s10344-014-0820-5.

Collado, L., Jara, R., and González, S. (2014). Description of Helicobacter valdiviensis sp. nov., an Epsilonproteobacteria isolated from wild bird faecal samples. *Int. J. Syst. Evol. Microbiol.* 64, 1913–1919. doi:10.1099/ijs.0.057141-0.

Dewar, M. L., Arnould, J. P. Y., Krause, L., Trathan, P., Dann, P., and Smith, S. C. (2014). Influence of Fasting during Moult on the Faecal Microbiota of Penguins. *PLOS ONE* 9, e99996. doi:10.1371/journal.pone.0099996.

Dominianni, C., Sinha, R., Goedert, J. J., Pei, Z., Yang, L., Hayes, R. B., et al. (2015). Sex, Body Mass Index, and Dietary Fiber Intake Influence the Human Gut Microbiome. *PLOS ONE* 10, e0124599. doi:10.1371/journal.pone.0124599.

Engström, B. E., Fermér, C., Lindberg, A., Saarinen, E., Båverud, V., and Gunnarsson, A. (2003). Molecular typing of isolates of Clostridium perfringens from healthy and diseased poultry. *Vet. Microbiol.* 94, 225–235. doi:10.1016/S0378-1135(03)00106-8.

Faith, D. P. (1992). Conservation evaluation and phylogenetic diversity. *Biol. Conserv.* 61, 1–10. doi:10.1016/0006-3207(92)91201-3.

Foster, G., Ross, H. M., Naylor, R. D., Collins, M. D., Ramos, C. P., Fernandez Garayzabal, F., et al. (1995). Cetobacterium ceti gen. nov., sp. nov., a new gram-negative obligate anaerobe from sea mammals. *Lett. Appl. Microbiol.* 21, 202–206.

Fox, J. G., Paster, B. J., Dewhirst, F. E., Taylor, N. S., Yan, L. L., Macuch, P. J., et al. (1992). Helicobacter mustelae isolation from feces of ferrets: evidence to support fecal-oral transmission of a gastric Helicobacter. *Infect. Immun.* 60, 606–611.

Geraci, J., Béchet, A., Cézilly, F., Ficheux, S., Baccetti, N., Samraoui, B., et al. (2012). greater flamingo colonies around the Mediterranean form a single interbreeding population and share a common history. *J. Avian Biol.* 43, 341–354. doi:10.1111/j.1600-048X.2012.05549.x.

Gillingham, M. A. F., Béchet, A., Courtiol, A., Rendón-Martos, M., Amat, J. A., Samraoui, B., et al. (2017). Very high MHC Class IIB diversity without spatial differentiation in the mediterranean population of greater flamingos. *BMC Evol. Biol.* 17, 56. doi:10.1186/s12862-017-0905-3.

Granada, C. E., Hasan, C., Marder, M., Konrad, O., Vargas, L. K., Passaglia, L. M. P., et al. (2018). Biogas from slaughterhouse wastewater anaerobic digestion is driven by the archaeal family Methanobacteriaceae and bacterial families Porphyromonadaceae and Tissierellaceae. *Renew. Energy* 118, 840–846. doi:10.1016/j.renene.2017.11.077.

Grond, K. (2017). Development and dynamics of gut microbial communities of migratory shorebirds in the Western Hemisphere.

Grond, K., Ryu, H., Baker, A. J., Domingo, J. W. S., and Buehler, D. M. (2014). Gastro-intestinal microbiota of two migratory shorebird species during spring migration staging in Delaware Bay, USA. *J. Ornithol.* 155, 969–977. doi:10.1007/s10336-014-1083-3.

Hird, S. M., Carstens, B. C., Cardiff, S. W., Dittmann, D. L., and Brumfield, R. T. (2014). Sampling locality is more detectable than taxonomy or ecology in the gut microbiota of the brood-parasitic Brown-headed Cowbird (Molothrus ater). *PeerJ* 2, e321. doi:10.7717/peerj.321.

Inglis, G. D., Hoar, B. M., Whiteside, D. P., and Morck, D. W. (2007). Campylobacter canadensis sp. nov., from captive whooping cranes in Canada. *Int. J. Syst. Evol. Microbiol.* 57, 2636–2644. doi:10.1099/ijs.0.65061-0.

Jumas-Bilak, E., Roudière, L., and Marchandin, H. (2009). Description of 'Synergistetes' phyl. nov. and emended description of the phylum 'Deferribacteres' and of the family Syntrophomonadaceae, phylum 'Firmicutes.' *Int. J. Syst. Evol. Microbiol.* 59, 1028–1035. doi:10.1099/ijs.0.006718-0.

Kapperud, G., and Rosef, O. (1983). Avian wildlife reservoir of Campylobacter fetus subsp. jejuni, Yersinia spp., and Salmonella spp. in Norway. *Appl. Environ. Microbiol.* 45, 375–380.

Kock, N. D., Kock, R. A., Wambua, J., Kamau, G. J., and Mohan, K. (1999). Mycobacterium avium-related epizootic in free-ranging less flamingos in Kenya. *J. Wildl. Dis.* 35, 297–300. doi:10.7589/0090-3558-35.2.297.

Krienitz, L., and Kotut, K. (2010). Fluctuating Algal Food Populations and the Occurrence of lesser flamingos (phoeniconaias Minor) in Three Kenyan Rift Valley Lakes1. *J. Phycol.* 46, 1088–1096. doi:10.1111/j.1529-8817.2010.00915.x.

Larsen, A. M., Mohammed, H. H., and Arias, C. R. (2014). Characterization of the gut microbiota of three commercially valuable warmwater fish species. *J. Appl. Microbiol.* 116, 1396–1404. doi:10.1111/jam.12475.

Lee, F. J., Rusch, D. B., Stewart, F. J., Mattila, H. R., and Newton, I. L. G. (2015). Saccharide breakdown and fermentation by the honey bee gut microbiome. *Environ. Microbiol.* 17, 796–815. doi:10.1111/1462-2920.12526.

Ley, R. E., Hamady, M., Lozupone, C., Turnbaugh, P. J., Ramey, R. R., Bircher, J. S., et al. (2008). Evolution of Mammals and Their Gut Microbes. *Science* 320, 1647–1651. doi:10.1126/science.1155725.

Li, Y., Xu, Q., Huang, Z., Lv, L., Liu, X., Yin, C., et al. (2016). Effect of Bacillus subtilis CGMCC 1.1086 on the growth performance and intestinal microbiota of broilers. *J. Appl. Microbiol.* 120, 195–204. doi:10.1111/jam.12972.

Liao, X. D., Ma, G., Cai, J., Fu, Y., Yan, X. Y., Wei, X. B., et al. (2015). Effects of Clostridium butyricum on growth performance, antioxidation, and immune function of broilers. *Poult. Sci.* 94, 662–667. doi:10.3382/ps/pev038.

Liu, H., Guo, X., Gooneratne, R., Lai, R., Zeng, C., Zhan, F., et al. (2016). The gut microbiome and degradation enzyme activity of wild freshwater fishes influenced by their trophic levels. *Sci. Rep.* 6, 24340. doi:10.1038/srep24340.

McMurdie, P. J., and Holmes, S. (2013). phyloseq: An R Package for Reproducible Interactive Analysis and Graphics of Microbiome Census Data. *PLOS ONE* 8, e61217. doi:10.1371/journal.pone.0061217.

Oksanen, J., Blanchet, F. G., Friendly, M., Kindt, R., Legendre, P., McGlinn, D., et al. (2018). *vegan: Community Ecology Package*. Available at: https://CRAN.R-project.org/package=vegan [Accessed May 25, 2018].

R Core Team (2018). *R: A Language and Environment for Statistical Computing*. Vienna, Austria: R Foundation for Statistical Computing Available at: https://www.R-project.org/.

Risely, A., Waite, D., Ujvari, B., Klaassen, M., and Hoye, B. (2017). Gut microbiota of a long-distance migrant demonstrates resistance against environmental microbe incursions. *Mol. Ecol.* 26, 5842–5854. doi:10.1111/mec.14326.

Risely, A., Waite, D. W., Ujvari, B., Hoye, B. J., and Klaassen, M. (2018). Active migration is associated with specific and consistent changes to gut microbiota in Calidris shorebirds. *J. Anim. Ecol.* 87, 428–437. doi:10.1111/1365-2656.12784.

Ryll, M., Christensen, H., Bisgaard, M., Christensen, J.-P., Hinz, K.-H., and Köhler, B. (2001). Studies on the Prevalence of Riemerella anatipestifer in the Upper Respiratory Tract of Clinically Healthy Ducklings and Characterization of Untypable Strains. *J. Vet. Med. Ser. B* 48, 537–546. doi:10.1111/j.1439-0450.2001.00471.x.

Ryu, H., Grond, K., Verheijen, B., Elk, M., Buehler, D. M., and Domingo, J. W. S. (2014). Intestinal Microbiota and Species Diversity of Campylobacter and Helicobacter spp. in Migrating Shorebirds in Delaware Bay. *Appl. Environ. Microbiol.* 80, 1838–1847. doi:10.1128/AEM.03793-13.

Shi, R., Yang, X., Chen, L., Chang, H., Liu, H., Zhao, J., et al. (2014). Pathogenicity of Shigella in Chickens. *PLOS ONE* 9, e100264. doi:10.1371/journal.pone.0100264.

Sommer, F., Ståhlman, M., Ilkayeva, O., Arnemo, J. M., Kindberg, J., Josefsson, J., et al. (2016). The Gut Microbiota Modulates Energy Metabolism in the Hibernating Brown Bear Ursus arctos. *Cell Rep.* 14, 1655–1661. doi:10.1016/j.celrep.2016.01.026.

Thomas, F., Hehemann, J.-H., Rebuffet, E., Czjzek, M., and Michel, G. (2011). Environmental and Gut Bacteroidetes: The Food Connection. *Front. Microbiol.* 2. doi:10.3389/fmicb.2011.00093.

Tsuchiya, C., Sakata, T., and Sugita, H. (2008). Novel ecological niche of Cetobacterium somerae, an anaerobic bacterium in the intestinal tracts of freshwater fish. *Lett. Appl. Microbiol.* 46, 43–48. doi:10.1111/j.1472-765X.2007.02258.x.

Vancanneyt, M., Vandamme, P., Segers, P., Torck, U., Coopman, R., Kersters, K., et al. (1999). Riemerella columbina sp. nov., a bacterium associated with respiratory disease in pigeons. *Int. J. Syst. Evol. Microbiol.* 49, 289–295. doi:10.1099/00207713-49-1-289.

Vanrompay, D., Ducatelle, R., and Haesebrouck, F. (1995). Chlamydia psittaci infections: a review with emphasis on avian chlamydiosis. *Vet. Microbiol.* 45, 93–119. doi:10.1016/0378-1135(95)00033-7.

Waldenström, J., Broman, T., Carlsson, I., Hasselquist, D., Achterberg, R. P., Wagenaar, J. A., et al. (2002). Prevalence of Campylobacter jejuni, Campylobacter lari, and Campylobacter coli in Different Ecological Guilds and Taxa of Migrating Birds. *Appl. Environ. Microbiol.* 68, 5911–5917. doi:10.1128/AEM.68.12.5911-5917.2002.

Wickham, H. (2016). *ggplot2: Elegant Graphics for Data Analysis*. Springer.

Zhang, J., Guo, Z., Lim, A. A. Q., Zheng, Y., Koh, E. Y., Ho, D., et al. (2014). Mongolians core gut microbiota and its correlation with seasonal dietary changes. *Sci. Rep.* 4, 5001. doi:10.1038/srep05001.

Zheng, A., Luo, J., Meng, K., Li, J., Bryden, W. L., Chang, W., et al. (2016). Probiotic (Enterococcus faecium) induced responses of the hepatic proteome improves metabolic efficiency of broiler chickens (Gallus gallus). *BMC Genomics* 17, 89. doi:10.1186/s12864-016-2371-5.
